# Genomic insights into the *Acidobacteria* reveal strategies for their success in terrestrial environments

**DOI:** 10.1111/1462-2920.14043

**Published:** 2018-03-12

**Authors:** Stephanie A. Eichorst, Daniela Trojan, Simon Roux, Craig Herbold, Thomas Rattei, Dagmar Woebken

**Affiliations:** ^1^ Division of Microbial Ecology, Department of Microbiology and Ecosystem Science Research Network “Chemistry Meets Biology”, University of Vienna Vienna Austria; ^2^ Department of Energy Joint Genome Institute Walnut Creek CA USA; ^3^ Division of Computational Systems Biology, Department of Microbiology and Ecosystem Science Research Network “Chemistry Meets Biology”, University of Vienna Vienna Austria

## Abstract

Members of the phylum *Acidobacteria* are abundant and ubiquitous across soils. We performed a large‐scale comparative genome analysis spanning subdivisions 1, 3, 4, 6, 8 and 23 (*n* = 24) with the goal to identify features to help explain their prevalence in soils and understand their ecophysiology. Our analysis revealed that bacteriophage integration events along with transposable and mobile elements influenced the structure and plasticity of these genomes. Low‐ and high‐affinity respiratory oxygen reductases were detected in multiple genomes, suggesting the capacity for growing across different oxygen gradients. Among many genomes, the capacity to use a diverse collection of carbohydrates, as well as inorganic and organic nitrogen sources (such as via extracellular peptidases), was detected – both advantageous traits in environments with fluctuating nutrient environments. We also identified multiple soil acidobacteria with the potential to scavenge atmospheric concentrations of H_2_, now encompassing mesophilic soil strains within the subdivision 1 and 3, in addition to a previously identified thermophilic strain in subdivision 4. This large‐scale acidobacteria genome analysis reveal traits that provide genomic, physiological and metabolic versatility, presumably allowing flexibility and versatility in the challenging and fluctuating soil environment.

## Introduction

The phylum *Acidobacteria* constitute an abundant and ubiquitous bacterial phylum typically found in soils and sediments. The widespread nature across members of this phylum in soils is clear; they have been detected in agricultural soils (Navarrete *et al*., 2013), forest soils (Štursová *et al*., 2012), peat soils (Pankratov, 2012), arctic tundra soils (Männistö *et al*., 2012a), desert soils (Kuske *et al*., 1997; Dunbar *et al*., 2002) and across many edaphically (defined here as of or relating to soil) diverse temperate soils (Jones *et al*., 2009). Not only are they ubiquitous, they also have a high relative abundance based on rRNA gene libraries (as high as 40% (range ca. 20%–40%) (Lipson and S. K. Schmidt, 2004; Janssen, 2006) and have a breadth of phylogenetic diversity similar to that of the Proteobacteria (Hugenholtz *et al*., 1998) spanning 26 subdivisions known to date (Barns *et al*., 2007).

Based on previous genomic and physiological investigations, many subdivision 1 and 3 strains have been described as versatile heterotrophs that grow optimally at low pH, produce copious amounts of extracellular material, harbour a low rRNA operon copy number suggesting an oligotrophic (more K‐selected) lifestyle, and contain both low‐specificity major facilitator superfamily and high‐affinity ABC‐type transporters (Ward *et al*., 2009; Rawat *et al*., 2012; Kielak *et al*., 2016). Some more striking physiologies such as iron reduction and/or fermentative growth (Liesack *et al*., 1994; Coates *et al*., 1999), phototrophy (Garcia Costas *et al*., 2012), along with the ability to grow under thermophilic conditions have been described in select strains from subdivision 4, 8, 10 and 23 (Izumi *et al*., 2012; Losey *et al*., 2013; Crowe *et al*., 2014; Stamps *et al*., 2014; Tank and Bryant, 2015a). Yet the vast majority of acidobacteria detected in soils based on culture dependent and independent approaches are members of subdivisions 1, 2, 3, 4, 5 and 6 (Janssen, 2006; Jones *et al*., 2009). Many strains in culture collections stem from these aforementioned ubiquitous subdivisions and were originally isolated from soil environments such as tundra soils (Männistö *et al*., 2012a), peatland soil (Pankratov *et al*., 2012; Dedysh *et al*., 2012), agricultural soil or meadow grassland soil (Eichorst *et al*., 2007; Eichorst and Kuske, 2012; Foesel *et al*., 2013), forest soils (Koch *et al*., 2008; Lladó *et al*., 2016) and subtropical soil (Huber *et al*., 2016). Therefore, it is particularly pertinent to better understand these environmentally relevant soil strains.

To that end, we sought to elucidate the potential ecophysiology of the soil acidobacteria by undertaking a large‐scale comparative acidobacterial genomic investigation comprising 24 genomes. Previous comparative genomic investigations on acidobacteria coupled with physiological‐based investigations have been limited to either three or six strains and have generated tremendous insights into their physiology (Ward *et al*., 2009; Rawat *et al*., 2012). The expansion of published genomes in the databases along with novel genomes from this study demanded an updated investigation, with a focus on the features in the genomes that could help explain their ubiquity and abundance in soil. More specifically, we investigated (i) if there are (any) systematic differences in gene content among these ‘subdivision’ classifications and/or environments from which the strains were isolated, (ii) events that shaped the genome structure of the strains, (iii) the genes that comprise the acidobacterial pan genome and (iv) what potential physiological features could allow for their ubiquity and prevalence across many soil environments.

## Results and discussion

### Phylogeny of investigated acidobacterial strains

**Figure 1:**
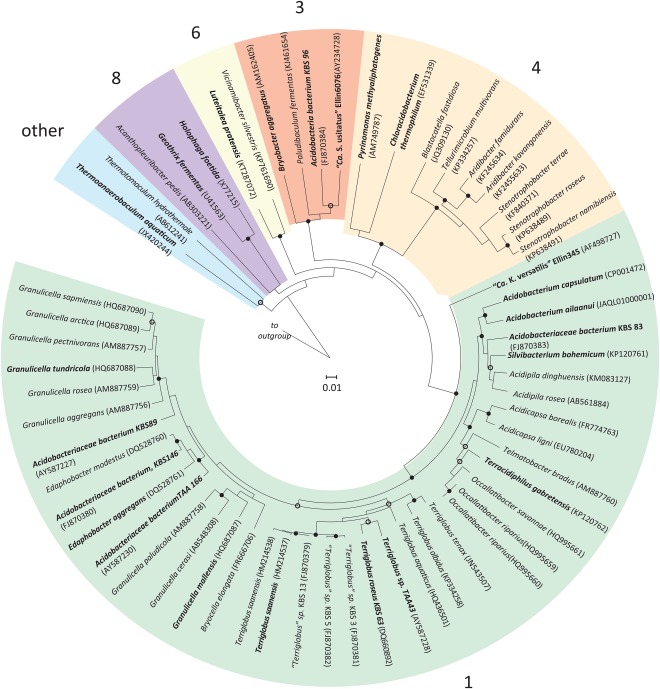
Neighbour‐joining tree of the acidobacterial 16S rRNA gene sequences retrieved from cultivated strains across subdivisions 1, 3, 4, 6, 8, 10 and 23. Strains investigated in this study are depicted in **bold**. Tree is displayed using the Interactive Tree of Life (Letunic and Bork, 2016). Number to the right of the shaded sections of the tree corresponds to the respective acidobacterial subdivision of the phylum. *Verrucomicrobium spinosum* (X90515) was used as an outgroup. The tree was bootstrapped 1000 times based on Jukes‐Cantor, and nodes with consensus support > 90% (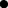) and > 70% (○) are displayed. The scale bar indicates 0.01 changes per nucleotide.

**Figure 2:**
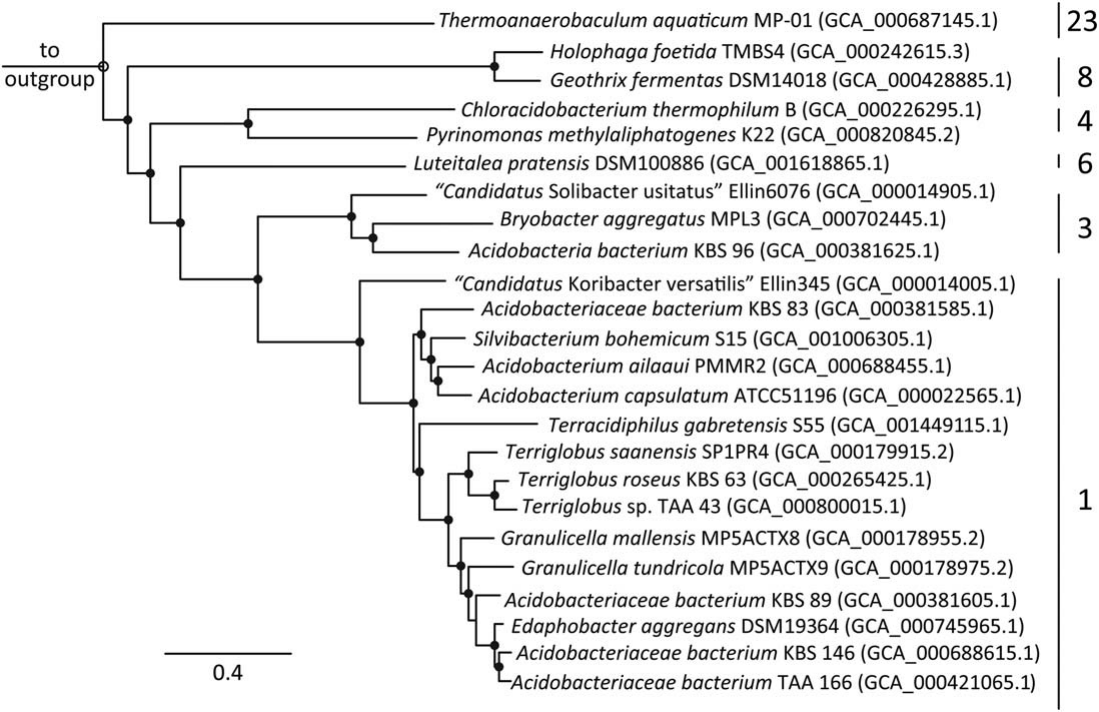
Bayesian inference phylogenomic tree based on a concatenated dataset of 43 universally conserved marker genes. Subdivisions are indicated to the right of the tree. The GenBank assembly accession number are depicted to the right of the strain. All nodes have ≥ 80% consensus support; more specifically nodes with > 99% (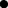) and > 94% (○) are displayed. The scale bar indicates 0.4 changes per nucleotide.

The genomes investigated in this study along with the references and abbreviations used can be found in Supporting Information Table S1. The general genome features of the new strains from this study are summarized in Supporting Information Table S2 and described in more detail in (Trojan *et al*., in preparation). Briefly, the genome sizes of the new strains ranged from 4.9 to 6.7 Mb with a genome GC content of 57%–60%. The number of estimated protein coding sequences (CDS) with predicted function ranged from 68% to 76%. Based on the 16S rRNA gene, the investigated genomes spanned subdivisions 1, 3, 4, 6, 8 and 23 (Fig. 1). There were at least two species in the genera *Terriglobus* and *Acidobacterium*, while the other genera were represented by one species. Nearly all genomes were at least 95% complete, with < 5% contamination based on CheckM analysis (Supporting Information Table S3) making them suitable for phylogenomic and comparative analyses (Parks *et al*., 2015). *Terriglobus roseus* KBS 63 was the one exception, with 100% completeness and 13.79% contamination; however, all marker genes (except one that was seen in multiple copies) were 100% identical at the amino acid level and the number of genes detected in the genome was not disproportionately high. This indicates a low level of population diversity rather than the presence of a contaminating organism and is not expected to affect phylogenomic and/or comparative analyses. The phylogenomic tree, based on 43 concatenated universally conserved marker genes, depicted a similar subdivision topology to the 16S rRNA gene tree, namely with clear distinctions across subdivisions 1, 3, 4, 6, 8 and 23 (Fig. 2, Fig. S1). Based on the phylogenomic tree, the phylum *Acidobacteria* branches as a sister clade to the *Aminicenantes* (formerly Candidate division OP8) (Supporting Information Fig. S1). *Aminicenantes* have been found in hydrocarbon‐impacted environments, marine habitats, aquatic, groundwater samples and terrestrial springs, typically having a higher relative abundance in environments characterized by low O_2_ concentrations, high temperatures and moderate to high salinity (Farag *et al*., 2014).

Subdivision 1 has the best representation in the phylogenomic tree with 15 genomes (Fig. 2). Within this subdivision, there is one main grouping consisting of three lineages: (i) *Silvibacterium* and *Acidobacterium*; (ii) *Terracidiphilus* and (iii) *Terriglobus*, *Granulicella* and *Edaphobacter*. There appears to be a major split with ‘*Candidatus* Koribacter versatilis’ Ellin345, as it is distinct from these lineages. The branching order of the phylogenomic tree relative to the 16S rRNA gene tree is fairly consistent with the exception of *Terracidiphilus*, which based on the 16S rRNA gene clusters with *Silvibacterium* and *Acidobacterium* (Supporting Information Fig. S1). Genomes stemming from the genus *Terriglobus* exhibited a monophyletic grouping consisting of *Terriglobus saanensis* SP1PR4, *T. roseus* KBS 63 and *Terriglobus* sp. TAA 43, and were most similar to a grouping consisting of *Granulicella* and *Edaphobacter* genomes. Interestingly, the two species in the *Granulicella* genus did not have a monophyletic nature based on the phylogenomic analysis, but formed a larger grouping with *Acidobacteriaceae bacteria* KBS 89, TAA 166 and KBS 146 along with *Edaphobacter aggregans* DSM19364. A similar pattern was observed in the 16S rRNA gene tree (Supporting Information Fig. S1). The other noted monophyletic genus cluster in subdivision 1 was the *Acidobacterium* genus, which encompassed *Acidobacterium capsulatum* ATCC51196 and *Acidobacterium ailaaui* PMMR2 (Fig. 2). This phylogenomic analysis provided additional insight into the phylogeny across members in the phylum *Acidobacteria*. It also suggests that genome information of additional strains will allow a better differentiation of the lineages and thus resolve the topology of the tree especially within subdivision 1.

### Average nucleotide and amino acid identity

The average nucleotide identity (ANI) and average amino acid identity (AAI) were compared across all investigated genomes (Supporting Information Fig. S2). The highest pairwise ANI values observed were between 70% and 78%, suggesting that all investigated genomes represented unique species (based on the proposed species definition encompassing shared gene content, which corresponds to the traditional 70% DNA‐DNA association standard (Konstantinidis and Tiedje, 2005a; Rodriguez‐R and Konstantinidis, 2014)). Genome clustering based on AAI was consistent with the subdivision‐level classification of the strains, and even within subdivisions it was largely consistent with known genera such as the *Terriglobus* cluster. The highest AAI ranged between 60% and 67% in subdivision 1 genomes, generating 3 clusters of high similarity: *Silvibacterium bohemicum* S15 and *A. ailaaui* PMMR2 with an AAI of 62%; *T. roseus* KBS 63 and *T.* sp. TAA 43 with an AAI of 67%; and *E. aggregans* DSM19364 having an AAI of 63% with *Acidobacteriaceae bacterium* KBS 89, 64% with *Acidobacteriaceae bacterium* TAA 166, and 68% with *Acidobacteriaceae bacterium* KBS 146. The clustering of these aforementioned genomes based on AAI could suggest that these are species of the same genus (Konstantinidis and Tiedje, 2005b).

### Genome structure and plasticity

Many factors can influence the composition, structure and plasticity of a genome, such as duplication events and genome rearrangements triggered by phages and other transposable and mobile elements. These events not only help to shape the genomes, but can also lead to the acquisition of new genetic potential (such as auxiliary metabolic genes) (Soucy *et al*., 2015). Genomes of acidobacterial strains isolated from soils typically harboured a larger genome size and proportion of paralogous genes, as compared to strains from 'other' environments such as hot springs and microbial mats (Supporting Information Fig. S3). Paralogous genes can stem from gene duplication events giving rise to potential genes with new function, as previously suggested for acidobacteria (Challacombe *et al*., 2011). Alternatively, this redundancy could be due to ecoparalogues (Sanchez‐Perez *et al*., 2008) – genes that have similar functions but are being expressed under different environmental conditions. Both features could be advantageous during resource fluctuations in soils and thus explain the larger proportion of paralogous genes compared to the genomes from ‘other’ environments such as hot springs, microbial mats and geothermal soils.

A total of 35 putative prophages were identified across 19 genomes (Table 1). The majority of genomes, in which prophages were detected, were from strains stemming from soils. This is in line with the notion that soils can have ca. 10^8^ virus particles per gram soil (Reavy *et al*., 2014) and harbour an extensive diversity and abundance of bacteriophage populations (Williamson *et al*., 2005). Prophages were not detected in *Acidobacteriaceae bacterium* KBS 146 (subdivision 1) and strains from ‘other’ environments (such as geothermal soils, hot springs, microbial mats), namely *Thermoanaerobaculum aquaticum* MP‐01 (subdivision 23), *Pyrinomonas methylaliphatogenes* K22 (subdivision 4), *Chloracidobacterium thermophilum* B (subdivision 4) and *A. ailaaui* PMMR2 (subdivision 1).

**Table 1:** Detected prophages and mobile genetic elements‐associated genes across the acidobacterial genomes.

		Activity		# of phage marker genes				Transposases & Integrases		
	# of detected prophages	Active^a^	Inactive/ decayed^b^	Phage‐Capsid	Terminase LSU	Portal	Phage Transposase & Integrase	Transposons‐ Transposase	Retro‐transposon R_integrase	Immunity Superinfection
**Subdivision 1**										
*Terriglobus roseus* KBS 63	1	1	ND	ND	1	1	8	1	ND	1
*Terriglobus* sp. TAA 43	3	2	1	ND	1	1	8	1	ND	ND
*Acidobacteriaceae bacterium* KBS 89	3	3	ND	2	ND	2	19	1	ND	ND
*Acidobacteriaceae bacterium* KBS 146	ND	ND	ND	ND	ND	1	9	1	1	ND
*Acidobacteriaceae bacterium* TAA 166	2	2	ND	2	1	2	24	3	5	1
*Acidobacteriaceae bacterium* KBS 83	2	1	1	1	ND	1	12	4	ND	1
*Acidobacterium capsulatum* ATCC51196	1	1	ND	ND	1	1	6	4	17	ND
*Acidobacterium ailaaui* PMMR2	ND	ND	ND	ND	ND	1	6	1	ND	1
*Granulicella tundricola* MP5ACTX9	1	1	ND	1	1	1	19	36	4	ND
*Granulicella mallensis* MP5ACTX8	2	2	ND	1	2	1	8	2	11	1
*‘Candidatus* Koribacter versatilis’ Ellin345	3	3	ND	ND	2	2	6	ND	1	ND
*Edaphobacter aggregans* DSM19364	4	2	2	2	ND	ND	57	64	3	ND
*Silvibacterium bohemicum* S15	1	1	ND	1	1	1	14	6	2	ND
*Terracidiphilus gabretensis* S55	1	1	ND	ND	ND	1	8	3	1	1
*Terriglobus saanensis* SP1PR4	2	2	ND	ND	1	1	10	1	6	2
**Subdivision 3**										
*Acidobacteria bacterium* KBS 96	4	4	ND	4	3	2	14	2	5	ND
*Bryobacter aggregatus* MPL3	1	1	ND	ND	1	ND	13	16	10	ND
*‘Candidatus* Solibacter usitatus’ Ellin6076	1	1	ND	ND	2	ND	31	14	32	ND
**Subdivision 4**										
*Chloracidobacterium thermophilum* B	ND	ND	ND	ND	ND	ND	4	2	ND	ND
*Pyrinomonas methylaliphatogenes* K22	ND	ND	ND	ND	ND	ND	2	ND	ND	ND
**Subdivision 6**										
*Luteitalea pratensis* DSM100886	1	1	ND	4	ND	ND	9	11	13	ND
**Subdivision 8**										
*Geothrix fermentans* DSM14018	1	ND	1	ND	ND	ND	5	3	1	ND
*Holophaga foetida* TMBS4	1	ND	1	ND	ND	ND	14	28	5	ND
**Subdivision 23**										
*Thermoanaerobaculum aquaticum* MP‐01	ND	ND	ND	ND	ND	ND	2	ND	ND	ND

‘ND’ – not detected. **a.** Indicate genomes where active prophages, defined as harbouring 1 or more virion‐associated genes, were detected. Multiple prophages were detected in the genomes of *Terriglobus* sp. TAA 43, *Acidobacteriaceae bacteria* KBS 89, TAA 166, KBS 83, *G. mallensis* MP5ACTX8, ‘*Ca.* K. versatilis Ellin345’, *E. aggregans* DSM19364, *T. saanensis* SP1PR4, and *Acidobacteria bacterium* KBS 96. **b.** Indicate genomes lacking virion‐associated genes, and likely representing decayed prophages from past infections.

Although *in silico* determination of the state of a temperate infection is challenging, as gene content alone cannot accurately reflect the activity of an integrated prophage, 29 out of these 35 putative prophages had a clear predicted prophage and harboured at least one virion‐associated gene (i.e., coding for the capsid, terminase, or portal, Table 1 under ‘Active’ and ‘# of phage marker genes’). This suggests that most of these predicted prophages may still have the genetic potential to complete a lytic cycle and, therefore, were considered as likely active. The remaining 6 putative prophages, identified across 5 genomes, did not display any virion‐associated gene, and were thus considered as likely representing degraded prophages (Table 1, under ‘Inactive/decayed’). These degraded prophages could be retained from past phage infections, which was previously observed and hypothesized to be one of the mechanisms by which ‘*Candidatus* Solibacter usitatus’ Ellin6076 acquired its large 9.9 Mb genome (Challacombe *et al*., 2011).

**Figure 3:**
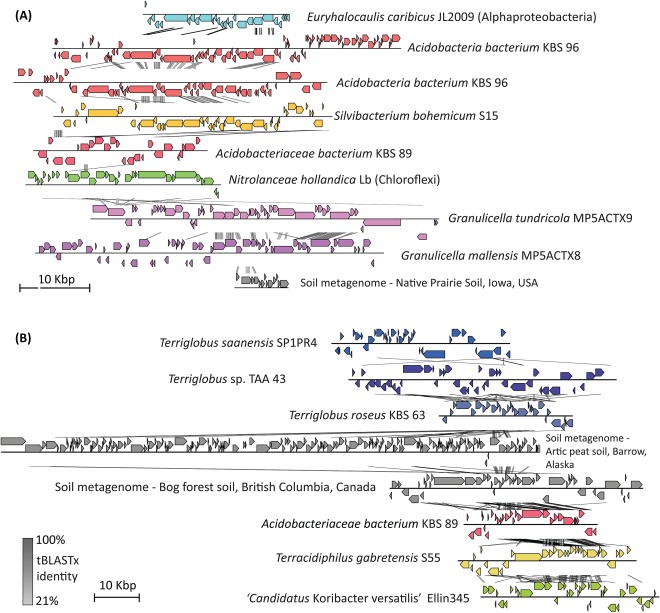
Two prophage clusters with high representation of the acidobacteria. A. Cluster 140 harbouring 6 scaffolds with acidobacteria (from top: (1) *Acidobacteria bacterium* KBS 96, ARMF01000004 G004DRAFT, scaffold00001_1_C4, 0 to 50000; (2) *Acidobacteria bacterium* KBS 96, ARMF01000009 G004DRAFT, scaffold00001_1_C9, 1290000 to 1335000; (3) *Silvibacterium bohemicum* S15, LBHJ01000004 contig_4, 395000 to 435000; (4) *Acidobacteriaceae bacterium* KBS 89, ARME01000012, G003DRAFT, scaffold00007_7_C, 50000 to 75000; (5) *Granulicella tundricola* MP5ACTX9 NC015064, 2275000 to 2325000; and (6) *Granulicella mallensis* MP5ACTX8, NC016631, 2110000 to 2160000). Additional contigs include *Euryhalocaulis caribicus* JL2009, contig00028; *Nitrolanceae hollandica* Lb, CAGS 00000000, contig 00252 to 1568; and a soil metagenome, contig 300000364, a_INPhiseqgaiiFebDRAFT_100851888. B. Cluster 153 with 6 scaffolds of acidobacteria (from top: (1) *Terriglobus saanensis* SP1PR4, NC014963, 3780000 to 3820000; (2) *Terriglobus* sp. TAA 43, JUGR01000001_M504DRAFT_scaffold00001_1_C, 80000 to 140000; (3) *Terriglobus roseus* KBS 63, NC018014, 150000 to 180000; (4) *Acidobacteriaceae bacterium* KBS 89, ARME01000010_G003DRAFT_scaffold00005_5_C, 60000 to 90000; (5) *Terracidiphilus gabretensis* S55, LAIJ01000009_contig10, 700000 to 740000; and (6) ‘*Candidatus* Koribacter versatilis’ Ellin345, NC008009, 5150000 to 5190000. Additional contigs include soil metagenome contig 3300001397_a_JGI20177J14857, 1000003 and 3300003218_a_JGI26339J46600, 10000010. The tBLASTx identity scores are given from 21% to 100%. The scale bar for each cluster is depicted in the respective label.

Many of the detected predicted prophages did not have a strong similarity to known phages and were instead identified due to a high concentration of unknown genes and a genome organization consistent with a phage genome. Based on the affiliation of the capsid‐related genes identified, these ‘acidobacterial prophages’ could nonetheless be tentatively affiliated to the order *Caudovirales*. None of the identified prophages clustered with sequenced phage isolates using a gene‐content based classification (corresponding to approx. genus‐level groupings) as described in (Lima‐Mendez *et al*., 2008). More specifically in this clustering, 12 prophages were singletons, 8 prophages clustered exclusively with other acidobacterial prophages, and the remaining 15 were associated with other prophages identified in publicly available microbial genomes or soil metagenomes (Roux *et al*., 2015b; Paez‐Espino *et al*., 2016). Two clusters were notable: one (Cluster 140) harbouring prophages from 5 different acidobacteria genomes (many of the genus *Granulicella*) alongside prophages from an Alphaproteobacterium (*Euryhalocaulis caribicus* JL2009) and a Chloroflexi (*Nitrolancea hollandica* Lb) with an average amino‐acid sequence identity (AAI) of 34.8% (Fig. 3A), and a second (Cluster 153) containing prophages from six acidobacterial genomes (many of the genus *Terriglobus*) and two phage genomes identified in soil microbial metagenomes with an average sequence identity of 37.8% (Fig. 3B). Given these amino‐acid identities, it suggests that these clusters are within the same subfamilies (Lavigne *et al*., 2008). At this time, further work is needed to better identify and characterize these potentially new viral groups associated with the phylum *Acidobacteria*.

There is evidence that select acidobacterial genomes could have been subject to multiple simultaneous viral infections. Eight genomes have traces of multiple prophages (i.e., multiple capsid, portal or terminase genes, Table 1, under ‘# of phage marker genes’), and 3 genomes (*Granulicella mallensis* MP5ACTX8, ‘*Ca*. K. versatilis’ Ellin345, and *Acidobacteria bacterium* KBS 96) contain 2 distinct and likely active prophages (in each case, both prophages include ≥ 40 genes and 1 or more virion‐associated genes). This level of polylysogeny is more typically associated with microbial pathogens, although recent work also suggested that lysogens may be favoured under conditions of highly variable bacterial densities (Touchon *et al*., 2016). This would be consistent with the soil environment, often limited in the availability of nutrients leading to sporadic growth and thus patchy distribution of soil organisms, which could promote polylysogeny in soil *Acidobacteria*. It is also noteworthy that 7 genomes (*T. roseus* KBS 63, *Acidobacteriaceae bacteria* KBS 83 and TAA 166, *G. mallensis* MP5ACTX8, *A. ailaaui* PMMR2, *T. saanensis* SP1PR4, *Terracidiphilus gabretensis* S55) include immunity to superinfection‐like genes (PFAM domain, Imm_superinfect) (Table 1). These genes are not necessarily in intact prophages suggesting that the bacteria might have acquired these genes from a decayed prophage and retained it to confer some level of immunity. Beyond the observation of multiple viral infections, the conservation of these prophage‐originating superinfection‐preventing genes is an additional indicator of the likely intense viral pressure experienced by soil *Acidobacteria*.

Further features that can shape bacterial genomes are transposable elements, which were also found in the investigated acidobacterial genomes based on the detection of transposase genes and putative retro‐transposons (Table 1, under ‘Transposases & Integrases'). These mobile genetic elements are more abundant than prophages in the acidobacterial genomes and in some cases, transposons and prophages are co‐localized in the same genome region, raising the possibility for gene transfer and/or recombination events between mobile genetic elements. Previous investigations notably detected various insertion sequence (IS) families across acidobacterial genomes (Challacombe and Kuske, 2012). Insertion sequences are short DNA sequences (< 2500 bp) that encode their own mobility proteins and can span multiple families and sub‐families (Mahillon and Chandler, 1998). Here, a general assessment of IS elements revealed that a broad range of IS elements were detected in select acidobacterial genomes (Supporting Information Table S4) highlighting that, in addition to prophages, these mobile genetic elements likely contribute substantially to soil *Acidobacteria* genome evolution.

Mobile elements and/or prophages can act as horizontal gene transfer agents and shuttle metabolically‐relevant genes across strains and species, which will impact the long‐term evolution and ecological success of their host (Frost *et al*. 2005). They can also encode in their own genome ‘auxiliary metabolic genes’ (i.e., genes expressed by the plasmid/phage), which in turn could help bacteria cope with adverse environmental conditions. The latter was documented previously in *Escherichia coli* (Wang *et al*., 2010) and bacteria in the deep ocean (Anantharaman *et al*., 2014; Roux *et al*., 2016), but remains mostly uncharacterized in soil systems. As such, this is a key and very interesting finding of this study and the first time prophage integration events were found to play a major role in the acidobacteria along with the detection and presences of putative active prophages and transposable elements in members of the *Acidobacteria* across many subdivisions. Downstream investigations should focus on whether these new mobile elements and/or prophage events act as a source of novel metabolic genes in the acidobacteria, possibly helping them to cope in adverse conditions.

### Genomic metabolic potential based on COG/NOGs

**Figure 4:**
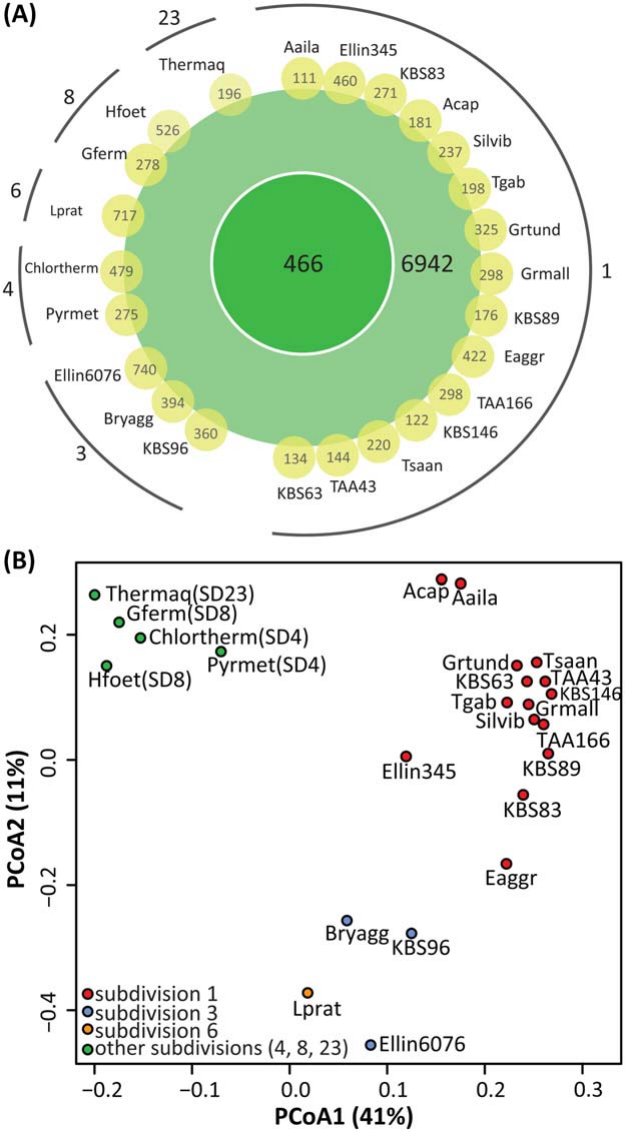
Analysis of the acidobacterial pan genome. A. The acidobacterial pan genome across strains in subdivisions 1, 3, 4, 8 and 23. The number of COGs/NOGs making up the core (dark green), variable (light green) and unique (yellow) genomes across the strains are depicted. Strains are displayed based on their phylogenetic clustering. B. The PCoA plot based on the COG/NOGs of the acidobacterial genomes calculated with the Bray–Curtis distance. Subdivisions are depicted in different colours and abbreviations for the strains can be found in Supporting Information Table S1.

The phylum *Acidobacteria* is subdivided into 26 subdivisions, yet it is unclear if these phylogenetic groupings correspond to different physiological capabilities among the strains. Of these 26 subdivisions, members of subdivision 1, 2, 3, 4, 5 and 6 are typically the most prevalent subdivisions in terrestrial environments (Janssen, 2006; Jones *et al*., 2009), suggesting that these members harbour a wide physiological range or core set of genes, which allow them to exploit various niches in soils. We expanded on previous comparative genome analysis on acidobacteria (Ward *et al*., 2009; Rawat *et al*., 2012) and revisited their genomic metabolic potential across 24 genomes. Our large‐scale acidobacterial pan‐genomic analysis revealed that the core genome of acidobacteria consisted of 466 COG/NOGs (Fig. 4A), which were distributed across multiple categories, with particular dominance in cellular processes and signalling [Cp], information storage and processing [Isp] and metabolism [Me] (Supporting Information Fig. S4). Approximately 9.8% of the acidobacterial core genome was classified as poorly characterized (Pc) (Supporting Information Fig. S4). The variable genomes (found in at least two genomes) comprised of 6,942 COG/NOGs (Fig. 4A), and they were also distributed across multiple categories. Across the 24 genomes, the number of unique COG/NOGs ranged from 111 (*A. ailaaui* PMMR2) to 740 (‘*Ca*. S. usitatus’ Ellin6076) (Fig. 4A).

Principle coordinate analysis of the COG/NOG categories across the 24 strains depicted clear groupings across strains in select subdivisions (Fig. 4B). The PcoA1 axis explained most of the variability (41%) and appeared to be due to the original isolation environment. Seventeen out of the nineteen strains that clustered were isolated from soil environments (such as meadow, agricultural or tundra soils – further description can be found in Supporting Information Table S1) spanning subdivision 1, 3 and 6 (Fig. 4B, red, blue & orange points), whereas strains from ‘other’ environments (such as hot springs, alkaline or thermophilic microbial mats, anoxic sediments and geothermal soil) clustered and belong to subdivision 4, 8 and 23 (Fig. 4B, green points). Permutational multivariate analysis of variance (PERMANOVA) revealed significant patterns due to the differences in subdivisions (*p* < 0.001) and environments (*p* < 0.006) illustrating that both factors helped to shape the distribution of the shared gene pool across the genomes.

The distinct clustering based on environment and subdivisions was further investigated to determine potential functional categories driving this structure. A chi‐square goodness of fit revealed that the functional categories did not have a similar distribution across genomes stemming from strains isolated of soil environment compared to ‘other’, suggesting that environment played a role in shaping the genomes. More specifically, the genomes of soil isolated strains had a significantly larger proportion of COG/NOGs in carbohydrate metabolism and transport (Me_G) (*p* < 0.003) and poorly characterized (Pc_S) (*p* < 0.0001), while ‘other’ genomes had a significantly larger proportion in energy metabolism (Me_C) (*p* < 0.005) and translation, ribosomal structure and biogenesis (Isp_J) (*p* < 0.003) (Supporting Information Fig. S5A).

The differential clustering between genomes in subdivision 1 and 3 was further investigated, as these harboured the largest number of genomes available for an initial subdivision‐level comparison. Some individual COG/NOGs were unique to respective subdivisions; for example, a copper resistance gene (NlpE, COG3015) and cyctochrome C biogenesis protein (COG4233) were only found in genomes of subdivision 1, while a tRNA isopentenyl‐2‐thiomethyl‐A‐37 hydroxylase (MiaE, COG4445) was only found in the genomes of subdivision 3. Although the distributions across functional categories were different between genomes of subdivision 1 and 3 based on chi‐square goodness of fit, no significant differences in the proportion across the individual categories were noted (Supporting Information Fig. S5B). We hypothesize that the differences between genomes of subdivision 1 and 3 stem from functionally related proteins in similar COG/NOG functional categories, suggesting that members of these subdivisions do not differ in the function itself, rather the manner in which they are able to perform the function. However, additional genomes, especially in subdivision 3, are necessary to strengthen this working hypothesis. The shared gene pool was further explored in members of subdivision 1 to ascertain if there was differential clustering on the genus‐level COG/NOGs; however, there was no clear clustering of the COGs/NOGs across strains in subdivision 1 (Supporting Information Fig. S6).

### Relationship to oxygen

The majority of the acidobacteria strains have been described as aerobes, capable of growing at 20% oxygen (O_2_) (vol/vol) and some capable of growth under reduced O_2_ (such as 1% or 2% O_2_) (Table 2) (Eichorst *et al*., 2011; Pankratov *et al*., 2012; Tank and Bryant, 2015a, 2015b). There is a, albeit small, collection of investigated acidobacteria that were reported to be either facultative (such as *A. ailaaui* PMMR2*, A. capsulatum* ATCC51196) (Pankratov *et al*., 2012; Myers and King, 2016) or strict anaerobes, namely in subdivision 8 (such as *Geothrix fermentans* DSM14018, *Holophaga foetida* TMBS4) (Liesack *et al*., 1994; Coates *et al*., 1999) and subdivision 23 (such as *T. aquaticum* MP‐01) (Losey *et al*., 2013). Given the prevalence of aerobic acidobacteria, we aimed at investigating whether some strains could have the genomic potential to respire O_2_ at atmospheric and sub‐atmospheric (or microoxic) concentrations. Therefore, we performed a detailed analysis on the presence and distribution of terminal oxidases with low‐ and high affinities for O_2_ across the investigated genomes.

**Table 2:** Catalytic subunits of high and low affinity terminal oxidases identified across the acidobacterial genomes and the strains’ previously reported relation to oxygen.

	No. of genes detected			
	Low‐affinity terminal oxidase	High‐affinity terminal oxidase		Reported growth regarding oxygen
	HCO^a^ Superfamily		*bd*‐type oxidase^b^	
	Type A	Type C (*cbb*_3_)		
**Subdivision 1**				
*‘Candidatus* Koribacter versatilis’ Ellin345	2	ND	1	Not reported
*Terriglobus saanensis* SP1PR4	5	ND	ND	Aerobic^d^
*Terriglobus roseus* KBS 63	2	1	ND	Aerobic^e^ Microaerobic^e^
*Terriglobus* sp. TAA 43	3	ND	ND	Aerobic^e^ Microaerobic^e^
*Granulicella mallensis* MP5ACTX8	3	ND	1	Aerobic^f^
*Granulicella tundricola* MP5ACTX9	3	ND	ND	Aerobic^f^
*Acidobacteriaceae bacterium* KBS 89	3	ND	ND	Aerobic^e^ Microaerobic^e^
*Acidobacteriaceae bacterium* KBS 146	1	ND	1	Microaerobic^g^
*Acidobacteriaceae bacterium* TAA 166	2	ND	1	Aerobic^e^ Microaerobic^e^
*Edaphobacter aggregans* DSM19364	5	2	ND	Aerobic^h^
*Terracidiphilus gabretensis* S55	2	ND	ND	Aerobic^i^
*Acidobacteriaceae bacterium* KBS 83	4	ND	ND	Aerobic^e^ Microaerobic^j^
*Acidobacterium capsulatum* ATCC51196	1	1	1	Aerobic^k^ Microaerobic^l^ Anaerobic^l^
*Silvibacterium bohemicum* S15	5	ND	2	Aerobic^m^
*Acidobacterium ailaaui* PMMR2	3	1	ND	Aerobic^l^ Microaerobic^l^ Anaerobic^l^
**Subdivision 3**				
*‘Candidatus* Solibacter usitatus’ Ellin6076	4	1	1	Not reported
*Acidobacteria bacterium* KBS 96	4	1	ND	Aerobic^j^ Microaerobic^j^ Anaerobic^j^
*Bryobacter aggregatus* MPL3	2	ND	ND	Aerobic^n^
**Subdivision 4**				
*Chloracidobacterium thermophilum* B	1	ND	1^c^	Microaerobic^o^
*Pyrinomonas methylaliphatogenes* K22	1	ND	1	Aerobic^p^
**Subdivision 6**				
*Luteitalea pratensis* DSM100886	3	1	1	Aerobic^q^
**Subdivision 8**				
*Holophaga foetida* TMBS4	1	ND	1	Anaerobic^r^
*Geothrix fermentans* DSM14018	1	ND	2	Anaerobic^s^
**Subdivision 23**				
*Thermoanaerobaculum aquaticum* MP‐01	1	ND	1^c^	Anaerobic^t^

*Classified as aerobic when growth was reported under 20% oxygen (atmospheric conditions). Classified as microaerobic when growing at 2% O_2_ (Eichorst, 2007; Eichorst *et al*., 2007), 0.2% O_2_ (Myers and King, 2016) or in the oxic‐anoxic interface of agar deep tubes (Tank and Bryant, 2015a, 2015b). Classified as anaerobic when growth was reported under 100% N_2_ (anoxic conditions).

**a.** HCO, heme‐copper oxygen oxidases,

**b.** *bd*‐type oxidase = cytochrome *bd* quinol oxidase. ‘ND’. not detected. For locus tags of the detected genes see Supporting Information Table S5.

**c.** Catalytic subunit‐like sequence with a cytochrome C domain.

**d.** Männistö *et al*. (2011);

**e.** Eichorst *et al*. (2007);

**f.** Männistö *et al*. (2012b);

**g.** Eichorst (2007);

**h.** Koch *et al*. (2008);

**i.** García‐Fraile *et al*. (2016);

**j.** Eichorst *et al*. (2011);

**k.** Kishimoto *et al*. (1991);

**l.** Myers and King, 2016;

**m.** Lladó *et al*., 2016;

**n.** Kulichevskaya *et al*., 2010;

**o.** Tank and Bryant (2015a, 2015b);

**p.** Crowe *et al*., 2014;

**q.** Vieira *et al*., 2017;

**r.** Liesack *et al*., 1994;

**s.** Coates *et al*., 1999;

**t.** Losey *et al*., 2013.

A total of 85 genes encoding for the catalytic subunits of terminal oxidases were detected and spanned superfamilies of both the heme‐copper oxidases (HCO) as well as the cytochrome *bd* quinol oxidase (not homologues to the superfamily of HCOs) across the investigated genomes (Table 2, Supporting Information Table S5). More specifically, all investigated genomes contained at least one homologue of the low‐affinity terminal oxidase belonging to the type A of the HCO superfamily (Pereira *et al*., 2001, 2008) (Table 2). Additionally, seven strains in subdivisions 1, 3 and 6 harboured the catalytic subunit for the high‐affinity *cbb*_3_ terminal oxidase (HCO type C) (*T. roseus* KBS 63, *E. aggregans* DSM19364, *A. capsulatum* ATCC51196, *A. ailaaui* PMMR2, ‘*Ca*. S. usitatus’ Ellin6076, *Acidobacteria bacterium* KBS 96 and *Luteitalea pratensis* DSM100886). The high‐affinity *bd*‐type was even more prevalent and widespread across the strains, as it was detected in 13 strains in subdivisions 1, 3, 4, 6, 8 and 23 (‘*Ca*. K. versatilis’ Ellin345, *G. mallensis* MP5ACTX8, *Acidobacteriaceae bacteria* KBS 146 and TAA 166, *A. capsulatum* ATCC51196, *S. bohemicum* S15, ‘*Ca*. S. usitatus’ Ellin6076, *C. thermophilum* B, *P. methylaliphatogenes* K22, *L. pratensis* DSM100886, *H. foetida* TMBS4, *G. fermentans* DSM14018 and *T. aquaticum* MP‐01) (Table 2). Only 3 strains harboured all three putative catalytic subunits of the high‐ and low‐affinity terminal oxidases: *A. capsulatum* ATCC51196, ‘*Ca*. S. usitatus’ Ellin6076 and *L. pratensis* DSM100886. Alternative oxidases (AOX) in the acidobacterial genomes were not detected.

Taken together this indicates that select acidobacteria, notably many strains originating from soils, harbour both putative high‐ and low‐affinity terminal oxidases thus having the genomic potential to respire O_2_ at atmospheric and sub‐atmospheric (or microoxic) concentrations. This genomic feature could provide a selective advantage in soils, since O_2_ is depleted in microenvironments such as soil aggregates and as soil moisture increases (Sexstone *et al*., 1985; Paul and Clark, 1996; van Elsas *et al*., 1997), and could help contribute to their success and ubiquity in soil. Yet beyond the function as a respiratory oxygen reductase, the high‐affinity terminal oxidases can accomplish additional physiological functions, such as O_2_‐scavenging ((Giuffrè *et al*., 2014) and references therein) and cyanide‐sensitive nitric oxide reductase activity (Elena *et al*., 2001). Likewise, the low‐affinity terminal oxidase can also play an important role in oxygen protection and detoxification (Ramel *et al*., 2013). These alternative functions could explain the existence of genes encoding for *bd‐*type (high‐affinity) and HCO type A (low‐affinity) oxidases in the genomes of anaerobic acidobacteria (*G. fermentans* DSM14018, *H. foetida* TMBS4 and *T. aquaticum* MP‐01*)*, as growth for those strains in the presence of O_2_ has not been demonstrated (Liesack *et al*., 1994; Coates *et al*., 1999; Losey *et al*., 2013).

Using a recent definition put forward by Morris and Schmidt, 2013, we propose that acidobacteria harbouring either HCO type C (*cbb_3_*‐type) or the *bd*‐type oxidase found in this study are microaerobes due to genomic detection of high‐affinity oxidases, presumably giving them the ability to respire O_2_ at microoxic concentrations. In support of this hypothesis, strains harbouring either the *cbb_3_* or *bd*‐type oxidase, such as *T. roseus* KBS 63, *A. capsulatum* ATCC51196, *Acidobacteriaceae bacteria* KBS 146 and TAA 166 and *Acidobacteria bacterium* KBS 96 were previously shown to grow under low O_2_ conditions (1% or 2% O_2_ v/v) ((Eichorst, 2007; Eichorst *et al*., 2007a; 2011), D. Trojan, S.A. Eichorst, unpublished work). This illustrates that some of these strains indeed have the capacity to respire at microoxic concentrations. Our microaerobe hypothesis of the remaining strains would need to be confirmed with growth‐based testing, in addition to testing the aforementioned alternative functions of these oxidases, which too would be advantageous in soil.

### Anaerobic respiration

Although not frequently described across typical soil acidobacteria (subdivision 1 and 3 strains), we, nevertheless, investigated the genomic potential for dissimilatory nitrate, nitrite, sulfate and sulfite reduction. Homologues of functional marker genes of dissimilarity sulfate reduction (*dsrABC*, *aprBA*) were not detected in any of these genomes. None of the strains seem to be able to perform complete denitrification to dinitrogen gas as several crucial marker genes were missing in all of the genomes (Supporting Information Table S6), yet there have been examples of this process being performed by a microbial consortium (Hayatsu *et al*., 2008). The *narG* operon, encoding for a membrane‐bound respiratory nitrate reductase (EC: 1.7.99.4) was detected in the genome of *G. fermentans* DSM14018 (Supporting Information Table S6), which is in accordance with growth‐based studies (Coates *et al*., 1999). The genome of *T. aquaticum* MP‐01 harboured the *napA* operon (Supporting Information Table S6) involved in dissimilatory nitrate reduction (e.g., Moreno‐Vivián *et al*., 1999; Morozkina and Zvyagilskaya, 2007), yet this capacity was not observed in growth‐based investigations (Losey *et al*., 2013). Although *G. mallensis* MP5ACTX8 was described to reduce nitrate to nitrite (Männistö *et al*., 2012b) and *Acidobacteria bacterium* KBS 96 was reported to reduce a small percentage (ca. 3%) of nitrate to nitrite (Eichorst *et al*., 2011), no dissimilatory nitrate reductases were identified in their genomes (Supporting Information Table S6). However, both genomes harboured putative assimilatory nitrate reductase genes (*nasA* genes), which has been suggested to be involved in dissimilatory nitrogen metabolism (e.g., Morozkina and Zvyagilskaya, 2007).

*NirS*, a marker gene for denitrification encoding a cytochrome *cd_1_*‐containing nitrite reductase, was not found in any genome. Interestingly, *Acidobacteria bacterium* KBS 96 harbours two copies of *nirK*, which encodes for the dissimilatory copper‐containing nitrite reductase (NiR) (Supporting Information Table S6). The genome of *Acidobacteria bacterium* KBS 96 encoded ORFs with copper‐binding motifs T1Cu (Cys‐Met‐His_2_) and T2Cu (His_3_‐H_2_O) along with the active site residues Asp and His, which are required for nitrate reducing activity (e.g., Antonyuk *et al*., 2005; Merkle and Lehnert, 2012; Antonyuk *et al*., 2013) suggesting that both CuNiR might be functional. However, at this time it is rather unlikely that *Acidobacteria bacterium* KBS 96 is indeed a denitrifying organism as no known nitric oxide reductase is encoded in the genome.

The genomes of *L. pratensis* DSM100886 (subdivision 6), *G. fermentans* DSM14018 (subdivision 8) and *H. foetida* TMBS4 (subdivision 8) harboured genes encoding for a dissimilatory nitrite reductase (*nrfHA)* that catalyses the reduction of nitrite to ammonia, in contrast to NirK or NirS that catalyse the conversion of nitrite to nitric oxide. This respiratory nitrite ammonification is not only described to contribute to energy conservation but also plays a major role in detoxification as it mediates the nitrosative stress response caused, e.g., by the presence of nitric oxide, nitrite or hydroxylamine (e.g., Zumft, 1997; Kern *et al*., 2011; Rajeev *et al*., 2015). Homologues for *norBC* (also termed cNOR) and *norZ* (also termed qNOR) were detected in the putative nitrate/nitrite‐ reducing strains (*L. pratensis* DSM100886, *G. fermentans* DSM14018*, H. foetida* TMBS4 and *T. aquaticum* MP‐01), but also in subdivisions 1 and 3 strains that did not harbour any dissimilatory nitrate/nitrite reductases (Supporting Information Table S6). The *norZ* gene has been found to date in both denitrifiers and non‐denitrifying strains, suggesting a function in detoxifying nitric oxide (Cramm *et al*., 1999; Büsch *et al*., 2002; Leang *et al*., 2003; Braker and Tiedje, 2003; Philippot, 2005) rather than in denitrification. Taken together, anaerobic respiration with either nitrate, nitrite, nitric oxide or nitrous oxide as alternative electron acceptors does not appear to be common among these investigated genomes.

Candidate homologues pertaining to iron reduction (*mtrA*/*mtrB* genes) were detected in several genomes (‘*Ca*. S. usitatus’ Ellin6076, *Acidobacteriaceae bacteria* KBS 83 and KBS 146, *Acidobacteria bacterium* KBS 96, *S. bohemicum* S15, *T. gabretensis* S55, *Bryobacter aggregatus* MPL3, ‘*Ca*. K. versatilis’ Ellin345, *E. aggregans* DSM19364, *G. mallensis* MP5ACTX8, *Granulicella tundricola* MP5ACTX9, *L. pratensis* DSM100886 and *T. saanensis* SP1PR4), but none of the genomes had the complete *mtr*/*omc* operons as described for dissimilatory iron reducers in *Shewanella* and *Geobacter* species (Leang *et al*., 2003; Wang *et al*., 2008). This suggests that these strains do not use dissimilatory Fe (III)‐reducing pathways similar to *Shewanella* and *Geobacter* species, in accordance with previous reports (Ward *et al*., 2009). Although *G. fermentans* DSM14018 and *T. aquaticum* MP‐01 were previously described as iron reducers (Coates *et al*., 1999; Nevin and Lovley, 2002; Losey *et al*., 2013), a cluster of c‐type cytochrome genes that seem to be related to the *omcA*/*mtrC* family was only detected in the genome of *G. fermentans* DSM14018 (G398DRAFT_01334–01338).

Few of the investigated strains have been described to be capable of fermentative growth (*T. aquaticum* MP‐01, *G. fermentans* DSM14018, *H. foetida* TMBS4, *A. ailaaui* PMMR2) (Liesack *et al*., 1994; Coates *et al*., 1999; Losey *et al*., 2013; Myers and King, 2016). We detected putative homologues of possible fermentation genes (Supporting Information Table S7); however, many of the enzymes show bifunctional activities and can be part of respiratory complexes, thus their true function remain to be confirmed with growth‐based studies. Taken together, the genome information is consistent with published growth‐based reports of these strains; neither anaerobic respiration nor fermentation are typically observed in strains of subdivision 1 and 3.

### Assimilatory nitrogen metabolism, nitrification and nitrogen fixation

Nitrogen (N) is an essential nutrient, and typically limiting for microorganisms in soils (Geisseler *et al*., 2010). As such, many soil microorganisms have the capacity of attaining N from mineral and organic forms – a feature that we explored in the acidobacteria. All investigated acidobacteria (except *T. aquaticum* MP‐01) harboured homologues for ammonia uptake, namely genes for the ammonium channel transporter family (*amtB* gene, TC: 1.A.11) and candidate genes for glutamate dehydrogenase (GDH, EC: 1.4.1.−2/3/4; encoded by *gdhA/gdh2*), glutamine synthetase (GS, EC: 6.3.1.2; encoded by *gltBD*) and glutamate synthase (GOGAT, EC: 6.3.1.2; encoded by *glnA*) (Supporting Information Table S6). Furthermore, the *amtB* gene was located in an operon with *glnK* (a signal transduction protein and member of the N regulatory protein P‐II, which is known to be involved in sensing the N status of the cell). It is hypothesized that *glnK* and *amtB* genes could interact directly via protein‐protein interaction serving multiple purposes, e.g., regulating ammonia assimilation during N starvation (Coutts *et al*., 2002; Blauwkamp and Ninfa, 2003). Putative genes encoding for an assimilatory nitrate reductase (NaR), nitrite reductase (NiR) and nitrate/nitrite porter (NNP, TC: 2.A.1.8) were found in *T. roseus* KBS 63, *G. mallensis* MP5ACTX8, *G. tundricola* MP5ACTX9, *A. ailaaui* PMMR2, ‘*Ca*. S. usitatus’ Ellin6076, *P. methylaliphatogenes* K22, *L. pratensis* DSM100886 and *Acidobacteria bacterium* KBS 96, *Acidobacteriaceae* bacteria KBS 146 and KBS 89 and *T.* sp. TAA 43. Only NNP genes were detected in ‘*Ca*. K. versatilis’ Ellin345, *T. saanensis* SP1PR4, *Acidobacteriaceae bacterium* TAA 166, *T. gabretensis* S55, *A. capsulatum* ATCC51196, *S. bohemicum* S15, *B. aggregatus* MPL3, *C. thermophilum* B, *H. foetida* TMBS4, *G. fermentans* DSM14018 and *T. aquaticum* MP‐01, whereas NiR genes were only detected in *Acidobacteriaceae bacterium* KBS 83 (Supporting Information Table S6). Genes for the amino acid‐polyamine‐organocation superfamily (TC: 2.A.3) as well as for the dicarboxylate/amino acid:cation symporter family (TC: 2.A.23) were abundant across the genomes. Additionally, the detection of chitinase genes could indicate that chitin might serve not only as a carbon source (Supporting Information Fig. S8) but also as a source of N.

We also found evidence that select acidobacterial genomes harbour putative homologues encoding for extracellular peptidases. Extracellular microbial peptidase activity is of great importance in soils, as it mobilizes ammonium along with other N cycling compounds (Bach *et al*., 2001). Many soil microorganisms express proteolytic activities (Bach *et al*., 2001) due to serine endopeptidases (EC: 3.4.21) (such as serine alkaline peptidases, subtilisins and subtilisin‐like peptidases) (Vranova *et al*., 2013), along with other bacterial extracellular peptidases (such as alkaline and neutral metalloendopeptidases (EC: 3.4.24) (Kalisz, 1988). Serine endopeptidases are often used as a marker enzyme for proteolysis activity in soil (Fuka *et al*., 2008; Brankatschk *et al*., 2011) and were widely distributed across most acidobacterial genomes (the secreted subtilisin‐like peptidase family S8 (sub)). In addition, the peptidases of family S53 were detected in all genomes except in those of the subdivision 8 members *H. foetida* TMBS4 and *G. fermentans* DSM14018 (Supporting Information Table S6). Homologues for various extracellular metalloendopeptidases were also detected across select genomes (Supporting Information Table S6). The presence of these putative peptidases in the acidobacterial genomes, especially ones originating from soils, could allow them to exploit different niches for N uptake during times of limitation.

Altogether, it seems possible that acidobacteria can use both inorganic (ammonia and/or nitrate/nitrite) and organic N (amino acids and other high molecular weight compounds) as their N sources. The abundance of transporters specific for amino acids, polyamines and organocations seemed to be especially high in the genomes stemming from terrestrial strains (Supporting Information Table S6), suggesting their potential to not only use inorganic but also organic N sources via mineralization. Soils have been reported to contain varying types and amounts of free amino acids (Monreal and McGill, 1985; Kielland, 1994), which could serve as a source of N. Although select strains have been reported to grow on ammonia, nitrate, nitrite and/or amino acids (Eichorst, 2007; Koch *et al*., 2008; Dedysh *et al*., 2012; Crowe *et al*., 2014; Tank and Bryant, 2015b; Myers and King, 2016; Vieira *et al*., 2017; D. Trojan and S.A. Eichorst, unpublished data), a more detailed growth‐based study is warranted, as previous work illustrated that while *C. thermophilum* B harboured putative genes for ammonium uptake, it was unable to grow solely on ammonium (Tank and Bryant, 2015b). In accordance with previous reports (Ward *et al*., 2009; Kielak *et al*., 2016), we could not find any genomic evidence that acidobacteria are capable of nitrification as neither *amoCAB* nor *nxrAB* genes were found in the currently available genomes, nor N_2_ fixation (*nif* genes) except in the genome of *H. foetida* TMBS4 where the *nif*HDKEN operon was detected (Supporting Information Table S6) (Kielak *et al*., 2016).

### Carbohydrate metabolism

The potential to utilize carbon (C) was previously investigated in a reduced number of genomes (Ward *et al*., 2009; Rawat *et al*., 2012), therefore, we wanted to revisit this analysis to encompass additional genomes. Typically 5%–9% of the CDSs across the acidobacterial genomes were dedicated to genes involved in the biosynthesis, transfer, breakdown and/or modification of carbohydrates with exceptions (Supporting Information Fig. S7). There were no visible patterns observed among the different subdivisions, but the genomes of *G. fermentans* DSM14018, *H. foetida* TMBS4 and *C. thermophilum* B appear to have a lower portion of their genomes dedicated to carbohydrate‐active enzymes, which is in line with their previously described physiology (Liesack *et al*., 1994; Coates *et al*., 1999; Tank and Bryant, 2015a). Interestingly these strains were not originally isolated from soils, which could suggest that terrestrial acidobacteria might dedicate more of their genome to carbohydrate metabolism (and presumably regulation of carbohydrate metabolism) potentially allowing for more flexibility and versatility.

Across all investigated genomes, 131 glycoside hydrolase (GH) families were found (Supporting Information Fig. S8), some of which are important enzymes for the breakdown of plant cell wall (Gilbert, 2010). The most prevailing GH families across the acidobacterial genomes were GH109 and GH74, both presumed to be involved in polymeric carbohydrate degradation. GH109 is an alpha‐N‐acetylgalactosaminidase (EC: 3.2.1.49) that acts on O‐linked oligosaccharides, which is typically found in chitin, bacterial peptidoglycan and lipopolysaccharide (Liu *et al*., 2007), whereas GH74 is an enzyme family that target the β‐1,4‐linkage of glucans (polysaccharide of glucose). Across the genomes, *H. foetida* TMBS4 did not harbour GH109 nor GH74, and there were few occurrences of GH109 in *T. aquaticum* MP‐01, *G. fermentans* DSM14018, and *C. thermophilum* B. Various GH families involved in the degradation of β‐glucosidic bonds, typical bonds found in cellulose (Berlemont and Martiny, 2013), were found across these investigated genomes. More specifically, GH5 was more consistently found across subdivision 1, 3, 4 and 6, while GH8, GH9, GH44 and GH12 were only detected in a few genomes of subdivision 1 (*T.* sp. TAA 43, ‘*Ca*. K. versatilis’ Ellin345, *T. gabretensis* S55, and *G. mallensis* MP5ACTX8) and once in subdivision 3 (GH9, ‘*Ca*. S. usitatus’ Ellin6076), supporting some of the previous GH family investigations (Ward *et al*., 2009). GH3 can encode for β‐glucosidase (an enzyme that hydrolyses the glycosidic bonds in oligosaccharides to glucose); it was found across all investigated genomes. Putative chitinases (GH18 and GH19 family) were previously identified in the genomes of *G. mallensis* MP5ACTX8, *G. tundricola* MP5ACTX9 and *T. saanensis* SP1PR4 (Rawat *et al*., 2012). Here we found CDSs mainly for GH18 across all subdivision 1 genomes, two genomes of subdivision 3 (‘*Ca*. S. usitatus’ Ellin6076 and *Acidobacteria bacterium* KBS 96), one genome of subdivision 4 (*P. methylaliphatogenes* K22), *L. pratensis* DSM100886 (subdivision 6) and *H. foetida* TMBS4 (subdivision 8), whereas CDSs for GH19 were only found in *G. fermentans* DSM14018, *P. methylaliphatogenes* K22, *L. pratensis* DSM100886, *T. saanensis* SP1PR4 and *E. aggregans* DSM19364 (Supporting Information Fig. S8).

In addition to the degradation of polymeric C compounds, select strains appear to have the possibility of anaplerotic carbon dioxide fixation. Homologues of phosphoenolpyruvate carboxylase and isocitrate dehydrogenase were detected across numerous genomes (Supporting Information Table S8) and were mostly, but not exclusively, distributed across the strains stemming from terrestrial environments. This feature could be advantageous for these strains as soils harbour pockets of elevated carbon dioxide. The presence of these CDSs suggests the possibility of carbon supplementation via anaplerotic pathways as was shown previously in *P. methylaliphatogenes* K22 (Lee *et al*., 2015). Select genomes also contained putative homologues for the *korAB* genes for carboxylation of succinyl CoA (via 2‐ketoglutarate ferredoxin oxidoreductase) and putative homologues for *porABDG* and/or *por/nifJ* for acetyl CoA carboxylation (pyruvate ferredoxin oxidoreductase) (Supporting Information Table S8). CDSs of RuBISco (EC: 4.1.1.39) were not detected.

The genomic data suggest that select strains have the potential to degrade plant polymeric C (such as cellulose and chitin) supporting previous findings (Ward *et al*., 2009; Rawat *et al*., 2012) and that they can also supplement intermediates in the citric acid cycles through anaplerotic pathways. Previous growth‐based work among many of the strains supports these findings with the utilization of a diverse collection of carbohydrates, such as plant polymeric C (Eichorst *et al*., 2011; Dedysh *et al*., 2012; Rawat *et al*., 2012).

### H_2_ scavenging

The thermophilic acidobacterial strain *P. methylaliphatogenes* K22 (subdivison 4) was identified to harbour the membrane‐bound hydrogen (H_2_)‐uptake [NiFe]‐hydrogenases group 1h (formely group 5) (hereafter referred to as ‘group 1h/5 hydrogenase’) (Greening, *et al*., 2015a). This strain was able to consume atmospheric levels of H_2_ due to the presence of this gene. Furthermore, the catalytic subunit was upregulated in stationary phase relative to the exponential phase suggesting that H_2_ uptake could be a strategy to survive periods of starvation (Greening, *et al*., 2015a). Although acidobacteria are detected in thermophilic environments, they are most abundant in more temperate soils (Jones *et al*., 2009). Select mesophilic strains, ‘*Ca*. S. usitatus’ Ellin6076 and *G. mallensis* MP5ACTX8 were previously identified to harbour the group 1h/5 hydrogenase (Constant *et al*., 2011; Greening, *et al*., 2015a).

**Figure 5:**
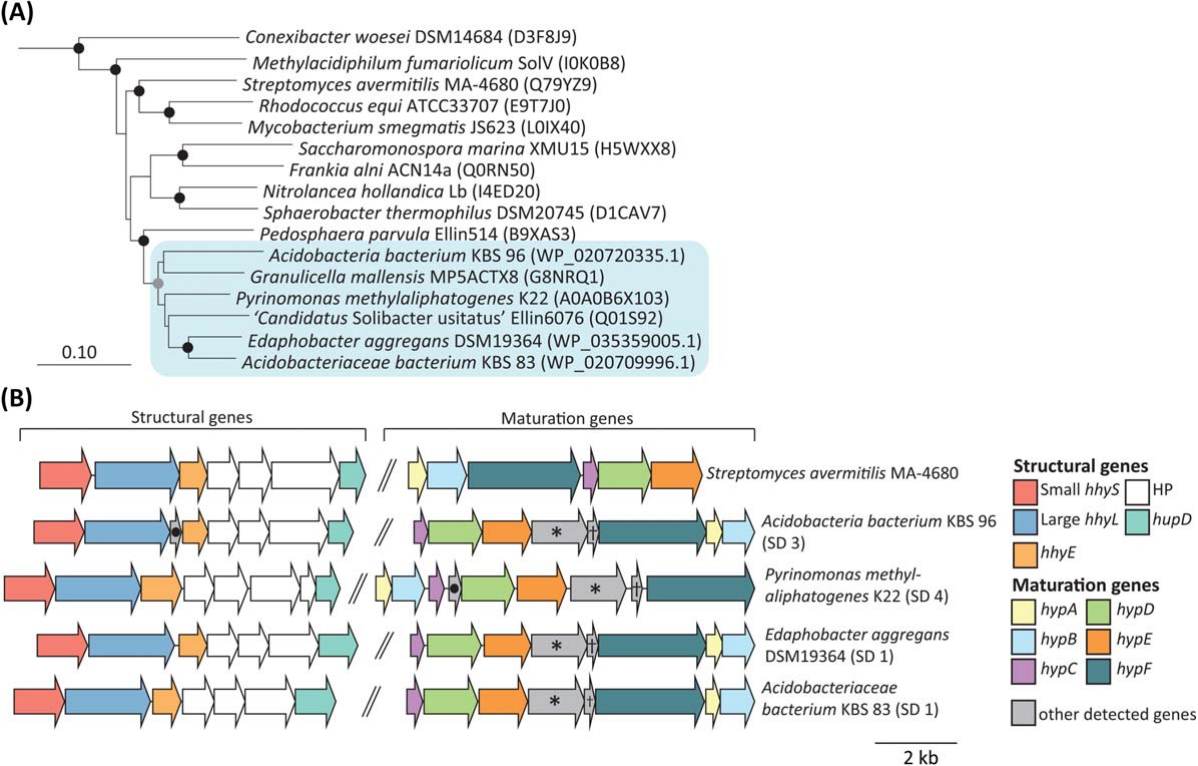
Distribution and gene organization of the group 1h/5 hydrogenases across the acidobacterial genomes. A. A neighbour‐joining tree based on the deduced amino acid sequence (ca. 560 amino acid positions) of the group 1h/5 [NiFe]‐hydrogenase large subunit. The tree was bootstrapped 1000 times, and the consensus support is displayed [> 95% (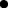) and > 90% (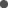)]. The outgroup (not shown) was the group 1 [NiFe]‐hydrogenase from *Desulfovibrio* sp. strain TomC (A0A0B1U1E4). B. The gene synteny of structural and maturation genes for the group 1h/5 [NiFe]‐hydrogenase across newly identified members of the phylum *Acidobacteria*. *Streptomyces avermitilis* MA‐4680 and *Pyrinomonas methylaliphatogenes* K22 are also shown for comparison. Unlabelled grey arrows depict other genes associated with the hydrogenase‐encoding locus: asterisks depict a putative phosphoheptose isomerase (*gmhA*), crosses depict a putative glutaredoxin 3 and circles depicts hypothetical proteins. Locus tags can be found in Supporting Information Table S9.

We expanded on this observation and identified two additional strains in subdivision 1 (*Acidobacteriaceae bacterium* KBS 83 and *E. aggregans* DSM19364) and one strain in subdivision 3 (*Acidobacteria bacterium* KBS 96) that harbour the group 1h/5 hydrogenase (Fig. 5A). Multiple genes predicted to be the necessary structural genes for this group 1h/5 hydrogenase, such as the small subunit (*hhyS*), large subunit (*hhyL*), a putative Fe‐S cluster (*hhyE*), a putative endopeptidase (*HupD*) as well as conserved hypothetical proteins (HP) (Fig. 5B) were identified in these aforementioned strains. All strains encode one copy of the large subunit of the hydrogenase, and the L1 and L2 signatures of this subunit were similar to the reported signatures of the group 1h/5 [NiFe]‐hydrogenase type (Constant *et al*., 2011) (Supporting Information Fig. S9). The large subunit across all identified acidobacteria share on average 75.8% deduced amino acid sequence identity to *Streptomyces avermitilis* ATCC31267. Furthermore, these acidobacteria strains form a distinct cluster, which is most similar to a strain in the phylum *Verrucomicrobia*, *Pedosphaera parvula* Ellin514 (average sequence identity of 77.5%) (Fig. 5A). Within these structural genes, three highly conserved proteins (Fig. 5A, ‘HP’) were detected, previously characterized as specific for this high‐affinity hydrogenase across all organisms encoding group 1h/5 hydrogenases, yet their function remains unknown (Greening, *et al*., 2015b). The structural subunits contain all residues to bind a [NiFe]‐centre for H_2_ cleavage (large subunit) and the three [4Fe4S] clusters for electron transfer (small subunit), suggesting that these hydrogenases could be functional in these acidobacterial strains. Multiple genes predicted to encode for maturation genes (*hypABCDEF*) (Fig. 5A), which are required for the function of hydrogenases (Greening, *et al*., 2015b), were also detected across the acidobacterial strains. The operon structure of these newly identified strains is somewhat conserved to *Streptomyces avermitilis* MA‐468 and *P. methylaliphatogenes* K22 (Fig. 5B). Conversely, the gene synteny of the previous identified acidobacteria, ‘*Ca*. S. usitatus’ Ellin6076 and *G. mallensis* MP5ACTX8 (Constant *et al*., 2011; Greening, *et al*., 2015a), were very distinct from *S. avermitilis* MA‐468 and *P. methylaliphatogenes* K22, namely the maturation protein genes for *G. mallensis* MP5ACTX8 were upstream of the catalytic subunits, while the catalytic subunits of ‘*Ca*. S. usitatus’ Ellin6076 were flanked by maturation proteins (Supporting Information Table S9).

Our analysis extends the detection of group 1h/5 hydrogenases to mesophilic acidobacteria in subdivision 1 and 3, yet the functionality of this hydrogenase across the strains still needs to be confirmed. Nevertheless, these data suggest that select members of the acidobacteria have the genes predicted to function as a group 1h/5 hydrogenases, which could provide them with a selective advantage for periods of starvation as hypothesized for *P. methylaliphatogenes* K22 (Greening, *et al*., 2015a). With this, the genomic potential to oxidize H_2_ at atmospheric levels using the group 1h/5 hydrogenase spans members in subdivisions 1, 3 and 4, which are typical subdivisions found in soils (Jones *et al*., 2009). Although it does not appear to be a widespread trait among all investigated acidobacterial genomes, it seems that the presence of these putative hydrogenases is not unique to a particular subdivision or environment type. A similar pattern can be seen in the Actinobacteria – select members in the Streptomycetes are believed to play a critical role in soil H_2_ consumption (Constant *et al*., 2010), yet not all Actinobacteria harbour a hydrogenase gene. Soils have been described as the major sink for H_2_, and soil bacteria are believed to control this consumption (Conrad, 1996; Greening, *et al*., 2015b). It has been hypothesized that this uptake could sustain approximately 6 x 10^7^ H_2_‐oxidizing soil bacteria per gram of soil, yet the identity of these potential H_2_‐oxidizing soil bacteria remains unknown (Conrad, 1996; Constant *et al*., 2011).

Group 1h/5 hydrogenases were not the only hydrogenases detected in the acidobacteria. We also found evidence for other [NiFe]‐hydrogenases in strains from subdivision 1, 3 and 23, such as group 1c [‘*Ca*. S. usitatus’ Ellin6076 (Acid_6926) and ‘*Ca*. K. versatilis’ Ellin345 (Acid345_4240)], group 1d [*T. aquaticum* MP‐01 (EG19_11475)], group 3c [*T. aquaticum* MP‐01 (EG19_05165)] and group 1f [*S. bohemicum* S15 (Ga0077217_103189) and *A. capsulatum* ATCC51196 (ACP_3053)]. Group 1f was also reported in a thermophilic strain in subdivision 1 (*A. ailaaui* PMMR2) (Myers and King, 2016). At this time, more physiological investigations across these non‐group 1h/5 hydrogenases are warranted.

## Conclusions and future outlook

Members of the phylum *Acidobacteria* are ubiquitous across terrestrial ecosystems worldwide, yet their ecological role(s) in these environments remains elusive. In this study, we sought to use comparative genomics to provide genomic insights into their ecophysiology. The vast majority of the analysed genomes originated from strains isolated from terrestrial environments, along with a few stemming from environments such as freshwater hot springs, freshwater mud, alkaline microbial mats, aquifer, acid mine drainage and geothermally heated microbial mat. Interestingly, these non‐terrestrial strains typically harboured a reduced genome size and proportion of paralogous genes, suggesting that these environments could be more stable and, therefore, metabolic versatility might not be necessary. In support of this conjecture, we found that the native isolation environment of the investigated strains along with the subdivision classification was a factor in shaping the distribution of genes across genomes. Genomes of acidobacterial strains isolated from soils were larger and typically harboured a more versatile repertoire of genes necessary for a changing environment, namely the genomic potential to use O_2_ at different concentrations, a diverse collection of carbohydrates, both inorganic (ammonia and/or nitrate/nitrite) and organic N (amino acids and other high molecular weight compounds) as their N sources and H_2_ at atmospheric concentrations. Mobile genetic elements, including temperate bacteriophages seem to have played a particular role in shaping the genomes of terrestrial acidobacterial genomes, yet it is unclear at this time if these events were vectors of horizontal gene transfer that introduced metabolism‐relevant genes.

Recent metagenomic investigations of environmental samples expanded our notion of the microbial tree of life, redefining the taxonomic definitions of phyla and give insights into potential physiologies. Although this study was limited to 24 genomes, the discussed findings are based on strains to which downstream growth‐based investigations can be performed to test working hypotheses. We believe that the appreciation of the discussed features in the potential physiological repertoire of soil acidobacteria will facilitate our understanding of this fascinating phylum and continue to bridge the knowledge gap between their ubiquity and their function in soil environments.

### Experimental procedures

#### Genome sequencing and assembly

Strains were grown on either a modified minimal medium or R2 medium as described previously (Eichorst, 2007; Eichorst *et al*., 2011; Kulichevskaya *et al*. 2010). Genomic DNA was isolated using a modified CTAB DNA extraction protocol as recommended by the DOE Joint Genome Institute (JGI).

The genome IDs and accession numbers can be found in Supporting Information Table S1. Sixteen genomes were publically available, while eight were newly sequenced in this study (Supporting Information Table S2). Generation of these genomes was done using either Pacific Biosciences (PacBio) (*Acidobacteriaceae bacterium* KBS 146 and *Bryobacter aggregatus* MPL3*)*, Illumina technology (Bennett, 2004) (*Acidobacteriaceae bacteria* KBS 89 and KBS 83, *Acidobacteria bacterium* KBS 96 and *Terriglobus* sp. TAA 43), a combination of Illumina (Bennett, 2004) and 454 technologies (Margulies *et al*., 2005) (*Terriglobus roseus* KBS 63) or a combination of PacBio and Illumina (*Acidobacteriaceae bacterium* TAA 166). All general aspects of library construction and sequencing performed at the JGI can be found at http://www.jgi.doe.gov.

Briefly, data were assembled with HGAP (version: 2.0.0) (Chin *et al*., 2013) or AllpathsLG (Gnerre *et al*., 2011) when either the PacBio or combination of PacBio and Illumina technologies were utilized respectively. All raw Illumina sequence data were passed through a filtering program (DUK), which removes known Illumina sequencing and library preparation artifacts (Mingkun L, Copeland A, Han J – in house script). The following steps were then performed for assembly of Illumina‐based libraries: (i) filtered Illumina reads were assembled using Velvet (version 1.1.04) (Zerbino and Birney, 2008), (ii) 1–3 kb simulated paired end reads were created from Velvet contigs using wgsim (https://github.com/ih3/wgsim), (iii) Illumina reads were assembled with simulated read pairs using Allpaths–LG (version r41043) (Gnerre *et al*., 2011). The 454 Titanium standard data and the 454 paired end data were assembled together with Newbler, version 2.3‐PreRelease‐6/30/2009. The Newbler consensus sequences were computationally shredded into 2 kb overlapping fake reads (shreds). Illumina sequencing data were assembled with VELVET, version 1.0.13 (Zerbino and Birney, 2008), and the consensus sequence were computationally shredded into 1.5 kb overlapping shreds. The 454 Newbler consensus shreds were integrated, the Illumina VELVET consensus shreds and the read pairs in the 454 paired end library using parallel phrap, version SPS – 4.24 (High Performance Software, LLC). The software Consed (Ewing *et al*., 1998; Ewing and Green, 1998; Gordon *et al*., 1998) was used in the following finishing process. Illumina data were used to correct potential base errors and increase consensus quality using the software Polisher developed at JGI (A. Lapidus, unpublished). Possible mis‐assemblies were corrected using gapResolution (C. Han, unpublished), Dupfinisher (Han and Chain, 2006), or sequencing cloned bridging PCR fragments with subcloning. Gaps between contigs were closed by editing in Consed, by PCR and by Bubble PCR (J‐F Cheng, unpublished) primer walks. A total of 40 additional reactions were necessary to close gaps and raise the quality of the finished sequence.

#### Genome annotation

Genes were identified using Prodigal (Hyatt *et al*., 2010), followed by a round of manual curation using GenePRIMP (Pati *et al*., 2010) for finished genomes and draft genomes in fewer than 20 scaffolds. The predicted CDSs were translated and used to search the National Center for Biotechnology Information (NCBI) nonredundant database, UniProt, TIGRFam, Pfam, KEGG, COG and InterPro databases. The tRNAScanSE tool (Lowe and Eddy, 1997) was used to find tRNA genes, whereas ribosomal RNA genes were found by searches against models of the ribosomal RNA genes built from SILVA (Pruesse *et al*., 2007). Other non–coding RNAs such as the RNA components of the protein secretion complex and the RNase P were identified by searching the genome for the corresponding Rfam profiles using INFERNAL (http://infernal.janelia.org). Additional gene prediction analysis and manual functional annotation was performed within the Integrated Microbial Genomes (IMG) platform developed by the Joint Genome Institute, Walnut Creek, CA, USA (http://img.jgi.doe.gov) (Markowitz *et al*., 2009).

#### General genome analysis and pan genome analysis

CheckM was used to estimate completeness and contamination of the genomes based on lineage‐specific markers (Parks *et al*., 2015). The phylogenomic tree was constructed using a concatenated data set of 43 universally conserved marker genes of ribosomal proteins and RNA polymerase domains and calculated by Bayesian inference running in PhyloBayes (Lartillot *et al*., 2009), the tree topology converged within 11 000 generations. The list of the universally conserved marker genes used in this study can be found in Parks *et al*., 2015. A comprehensive set of outgroups was included. General genome statistics (such as genome size and percent of paralogous genes) was determined using the JGI IMG database (Markowitz *et al*., 2009). Average nucleotide identity (ANI) was calculated using MiSI (Varghese *et al*., 2015). Average amino acid identity (AAI) (Konstantinidis and Tiedje, 2005b) was calculated directionally between each genome pair using amino acid sequences predicted by Prodigal (Hyatt *et al*., 2010). Target amino acid sequences were required to align over 70% of the query sequence with at least 30% identity. For each directional calculation, the percent identity was normalized by query gene length and the reported AAI is the average between each directional AAI calculation.

The neighbour‐joining algorithm in ARB was used for the generation of the acidobacteria phylogenetic tree (Fig. 1) of the 16S rRNA gene using sequences (ca. 1400 bp) obtained from cultivated representatives from the SILVA_Living Tree (v106), publically available genomes and from genomes of this study. Sequences were aligned by the SINA online tool (Pruesse *et al*., 2012). The Neighbour‐Joining, Jukes‐Cantor substitution model in the Geneious software v10.0.8 (Auckland, New Zealand) was used for bootstrapping analysis. Displayed bootstrap values are based on 1000 bootstrap iterations. Tree is displayed using the Interactive Tree of Life (Letunic and Bork, 2016).

For evolutionary and functional analysis, COG/NOGs were predicted with NCBI COGSoft (Kristensen *et al*., 2010), based on the eggNOG database (4.0 release) (Powell *et al*., 2014). Local COG/NOG assignments in proteins were encoded in a custom database according to the COGSoft requirements. The acidobacterial pan genome analysis was based on the distribution of the predicted COG/NOGs: core genomes was defined as being present across all investigated genomes; variable genomes need to be present in 2 or more genomes and unique genomes were COG/NOGs unique to the respective genome. Permutational multivariate analysis of variance (PERMANOVA) was performed using the adonis function and PcoA using the vegdist function, both found in the vegan package in R (https://www.R-project.org). To ascertain if there were any functional differences in the genomic potential of the acidobacteria across environments and subdivision (1 vs. 3), the distribution of the COG functional categories were compared using a chi‐square test for goodness of fit with a Bonferroni's error rate adjustment (Samuels, 1989) and the average within each category was compared using analysis of variance in the R package (aov function) (https://www.R-project.org).

#### Aerobic and anaerobic respiration, fermentation, nitrogen metabolism, carbon metabolism

THE IMG website was used for comparative genome analyses and comparing function profiles across the genomes. The Pfam, COG, KOG, TIGRFam, KEGG and InterPro databases were used to identify marker genes and key enzymes of interest. Additionally, the gene neighbourhoods of detected genes were inspected manually. Locus tags for the genes identified across the acidobacterial genomes encoding for cytochrome terminal oxidases can be found in Supporting Information Table S5, anaerobic respiration and fermentation (Supporting Information Tables S6 and S7 respectively), heterotrophic carbon dioxide fixation (Supporting Information Table S8) and those for the identified marker genes involved in nitrogen metabolism in Supporting Information Table S6. The CDSs involved in carbohydrate metabolism were identified using the database for automated Carbohydrate‐active enzyme annotation (dbCAN) (Yin *et al*., 2012).

#### Detection of group 1h/5 hydrogenases

The detection of the group 1h/5 large and small catalytic subunit genes was initially detected by keyword searches and ProteinBLAST in the JGI IMG database (Markowitz *et al*., 2009) and confirmed with the HydDB (Søndergaard *et al*., 2016). Further phylogenetic analysis of the large and small catalytic subunits along with annotation of the conserved L1 and L2 signatures (Constant *et al*., 2011) were done in ARB (Ludwig *et al*., 2004). The gene synteny of the structural and maturation genes was visualized with the GenoPlotR package in R (Guy *et al*., 2010). Locus tag for the genes identified across the acidobacterial genomes encoding for group 1h/5 [NiFe]‐hydrogenases can be found in Supporting Information Table S9.

#### Detection of prophages

The genomes were screened for the presence of prophages using VirSorter (Roux *et al*., 2015a) and PHASTER (Arndt *et al*., 2016). Prophage predictions were manually curated to remove false‐positives and verify prophage ends, leading to a total of 35 prophages identified. Predicted proteins from these prophages (extracted from VirSorter output) were affiliated against RefSeq Virus v70 (O'Leary *et al*., 2016) using blastp (Altschul *et al*., 1997), and against PFAM v27 (Finn *et al*., 2014) using HMMER 3 (Finn *et al*., 2011).

Prophages were classified as active when they included at least 1 gene Involved in virion formation (i.e., coding for a capsid, terminase, or portal protein). The hypothesis is that only prophages lacking these genes would be likely defective, i.e., not able to switch to a lytic cycle, and thus would stay inserted into the host genome until they progressively decay. It has to be noted, however, that such prophages lacking a virion machinery are not necessarily defective, but could also represent satellite phages, while some prophages encoding capsid‐related genes have been shown to lack the ability to self‐replicate (Casjens, 2003). Hence, we chose to designate prophages only as ‘predicted’ active or inactive.

A genome‐based classification of these prophages, based on protein clustering and network clustering as done previously (Lima‐Mendez *et al*., 2008; Roux *et al*., 2015a, 2015b), was performed using vContact (https://bitbucket.org/MAVERICLab/vcontact, sig score ≥ 2, default parameters otherwise). Briefly, viral genomes (complete or partial, from free‐living viruses or integrated prophages) are clustered into approximately genus‐level groups based on a shared genes network. The database used here included complete bacteria and archaea virus genomes from RefSeq v70, the VirSorter Curated Dataset (Roux *et al*., 2015a, 2015b), and the Earth's Virome Dataset (Paez‐Espino *et al*., 2016). Easyfig (Sullivan *et al*., 2011) was used to generate genome comparison plots with members of two clusters including five Acidobacteria prophages each (clusters 140 and 153, Fig. 3). Pairwise Amino‐acid identity levels between (pro)phages were calculated using the aai tool from the enveomics package (Rodriguez‐R and Konstantinidis, 2016).

## Conflict of Interest

No conflicts of interest are declared.

## Supporting information

Additional Supporting Information may be found in the online version of this article at the publisher's web‐site:

Materials and methods for Fig. S1.
**Table S1.** Genomes used in this study including isolation source of strains and associated references.
**Table S2.** General genome features across new genomes from this study.
**Table S3.** Genome completeness, contamination and strain heterogeneity across all investigated genomes based on CheckM
**Table S4.** Preliminary assessment of putative insertion sequence (IS) element families across the acidobacterial genomes. Abbreviations for the genomes can be found in Table S1. Numbers represent complete/partial/pseudogene/unknown IS elements. Analysis was performed using the ISsaga – IS Semiautomatic genomic annotation website (http://issaga.biotoul.fr/issaga_login.php?type=2). Putative IS elements need to be manually curated.
**Table S5.** Locus tags for the genes identified across the acidobacterial genomes encoding for the catalyticsubunit of respiratory oxygen reductases (cytochrome terminal oxidases). Locus tags for each gene starts with the ID given in column “locus tag start”, followed by the number in the respective column.
**Table S6.** Marker genes identified for nitrogen metabolism across acidobacterial genomes. Columns provide the function ID (EC or TC number), product (enzyme, transporter) name, gene name and locus numbers for each genome represented by its strain name.
**Table S7.** Locus tags across the acidobacterial genomes encoding for putative genes involved in fermentation pathways.
**Table S8.** Locus tags for the genes identified across the acidobacterial genomes encoding for genes involved in heterotrophic carbon dioxide fixation. The complete locus tags for each genome are listed.
**Table S9.** Locus tag for the putative genes along with genome ID identified across the acidobacterial genomes associated with the group 1h/5 [NiFe]‐hydrogenases. Locus tags for each gene starts with “locus tag start”, followed by the number in the respective column.
**Fig. S1.** Panel a depicts an acidobacterial 16S rRNA gene phylogenetic tree (ca. 1,248 nucleotides) of genomes retrieved in this study (8 taxa) and from publicly available genomes of cultivated strains (16 taxa), as inferred by maximum likelihood (RAxML), using the general time‐reversible substitution model under the gamma model of rate heterogeneity (GTRGAMMA). Bootstrap support values (1000 iterations) are given on the branches of the tree. Genome assembly‐ and accession numbers, coordinates and accession numbers of the 16S rRNA genes are given in brackets. Numbers to the right of the tree correspond to the acidobacterial subdivisions. Scale bar indicates estimated nucleotide substitution per site. The root was placed on the branch leading to the *Actinobacteria*, belonging to the *Terrabacteria*. Panel b depicts the comprehensive phylogenomic tree (the part marked in grey is shown in Fig. 2) by Bayesian inference based on a concatenated dataset of 43 universally conserved marker genes. The scale bar indicates 0.4 changes per nucleotide. Additional details can be found in Supporting Information.
**Fig. S2.** Average nucleotide identity (panel a) and average amino acid identity (panel b) clustering analysis across the acidobacteria genomes. The heatmap was generated in the R package (ggplot2). Colour scale bar represents percent identity.
**Fig. S3.** Distribution of genome size (panel a) and percent of paralogous genes (panel b) across genomes stemming from ‘soils’ and ‘other’ environments. The percent of paralogous genes were normalized to gene count. The definition of ‘soil’ vs. ‘other’ genomes can be found in Supporting Information Table S1. Data were obtained from the IMG JGI website.
**Fig. S4.** Gene content by COG functional categories within the acidobacterial core genome. Each colour represents a different genome. Functional categories are grouped by metabolism (Me), cellular processes (Cp), information storage and processing (Isp) and poorly characterized (Pc).
**Fig. S5.** Boxplot distribution of COG functional categories across environments (panel a) and subdivision 1 and 3 genomes (panel b). COG functional categories are grouped by cellular processes (Cp), information storage and processing (Isp), metabolism (Me) and poor characterized (Pc). The description of the sub‐categories can be found in Supporting Information Fig. S3 and definition of ‘soil’ vs. ‘other’ genomes can be found in Supporting Information Table S1. Asterisks depict significant differences based on a chi‐square goodness of fit.
**Fig. S6.** Principal coordinate analysis plot based on the COG/NOGs of the genomes of genera from subdivision 1 based on the Bray–Curtis distance. The listed abbreviations for the genomes can be found in Supporting Information Table S1. Genera are depicted in different colours.
**Fig. S7.** Distributions of CDSs encoding for carbohydrate‐active enzymes based on the database dbCAN (http://csbl.bmb.uga.edu/dbCAN/index.php), specifically depicting polysacharide lyases, glycosyl transferases, glycoside hydrolases, carbohydrate‐binding modules, carbohydrate esterase and auxiliary activities. ‘Other’ denotes a sum of CDSs encoding for cohesion, dockerin and S‐layer.
**Fig. S8.** Distributions of glycoside hydrolases (GH) among the acidobacterial genomes, separated by subdivisions based on the dbCAN (http://csbl.bmb.uga.edu/dbCAN/index.php). The GH family number is depicted on the right‐hand side. Scale bar depicts the Z‐scores for each GH family; the darker the colour, the more putative CDSs in each respective genome was identified.
**Fig. S9.** Consensus L1 and L2 signature in the large subunit of the group 1h/5 [NiFe]‐hydrogenase across the 6 acidobacterial strains. Images were generated using WebLogo (http://weblogo.berkeley.edu). Reference: Crooks, G. E., Hon, G., Chandonia, J.‐M., and Brenner, S. E. (2004). WebLogo: a sequence logo generator. *Genome Res* **14**:1188–1190.
