# Supplementary material for: Genomic insights into the Acidobacteria reveal strategies for their success in terrestrial environments
Source: Environ Microbiol. 2018 Mar 12;20(3):1041–63. doi: 10.1111/1462-2920.14043 (PMC5900883; doi:10.1111/1462-2920.14043)
Supplement: Supplementary file 1 — Materials and methods for Fig. S1. Table S1. Genomes used in this study including isolation source of strains and associated references. Table S2. General genome features across new genomes from this study. Table S3. Genome completeness, contamination and strain heterogeneity across all investigated genomes based on CheckM Table S4. Preliminary assessment of putative insertion sequence (IS) element families across the acidobacterial genomes. Abbreviations for the genomes can be found in Table S1. Numbers represent complete/partial/pseudogene/unknown IS elements. Analysis was performed using the ISsaga – IS Semiautomatic genomic annotation website (http://issaga.biotoul.fr/issaga_login.php?type=2). Putative IS elements need to be manually curated. Table S5. Locus tags for the genes identified across the acidobacterial genomes encoding for the catalyticsubunit of respiratory oxygen reductases (cytochrome terminal oxidases). Locus tags for each gene starts with the ID given in column “locus tag start”, followed by the number in the respective column. Table S6. Marker genes identified for nitrogen metabolism across acidobacterial genomes. Columns provide the function ID (EC or TC number), product (enzyme, transporter) name, gene name and locus numbers for each genome represented by its strain name. Table S7. Locus tags across the acidobacterial genomes encoding for putative genes involved in fermentation pathways. Table S8. Locus tags for the genes identified across the acidobacterial genomes encoding for genes involved in heterotrophic carbon dioxide fixation. The complete locus tags for each genome are listed. Table S9. Locus tag for the putative genes along with genome ID identified across the acidobacterial genomes associated with the group 1h/5 [NiFe]‐hydrogenases. Locus tags for each gene starts with “locus tag start”, followed by the number in the respective column. Fig. S1. Panel a depicts an acidobacterial 16S rRNA gene phylogenetic tree (ca. 1,248 nucleotides) [file EMI-20-1041-s001.pdf]

Supplemental Information.

Materials and methods for Figure S1.

The acidobacterial 16S rRNA genes were extracted from publicly available genomes and from genomes from this study and were aligned by the SINA online tool (Pruesse *et al.*, 2012). For the phylogenetic inference of the 16S rRNA gene, additional outgroup sequences were downloaded from the SILVA SSU 128 database (Quast *et al.*, 2013). The final alignment contained 48 sequences with a minimum sequence length of 1248 nucleotides. The alignment was inspected in SeaView version 4.6.1 (Gouy *et al.*, 2010). A maximum likelihood (ML) based inference of the 16S rRNA gene tree was calculated by RAxML version 8.2.9 (Stamatakis, 2014), using the general time-reversible substitution model under the gamma model of rate heterogeneity (GTRGAMMA). 50 ML trees were generated and 1000 bootstrap replicates were computed to draw bipartitions on the best ML tree. The outgroup consists of a diverse collection of 16S rRNA gene sequences, including deltaproteobacterial sequences and Aminicenantes/OP8 sequences.

References:

Gouy, M., Guindon, S., and Gascuel, O. (2010) SeaView Version 4: a multiplatform graphical user interface for sequence alignment and phylogenetic tree building. *Molecular Biology and Evolution* **27**: 221–224.

Pruesse, E., Peplies, J., and Glockner, F.O. (2012) SINA: Accurate high-throughput multiple sequence alignment of ribosomal RNA genes. *Bioinformatics* **28**: 1823–1829.

Quast, C., Pruesse, E., Yilmaz, P., Gerken, J., Schweer, T., Yarza, P., *et al.* (2013) The SILVA ribosomal RNA gene database project: improved data processing and web-based tools. *Nucleic Acids Res.* **41**: D590–D596.

Stamatakis, A. (2014) RAxML version 8: a tool for phylogenetic analysis and post-analysis of large phylogenies. *Bioinformatics* **30**: 1312–1313.

Table S1. Genomes used in this study including isolation source of strains and associated references.

	Reference	Environment	Abbreviations used in this study	GenBank Accession Number	NCBI Genome ID	GenBank Assembly Accession	IMG Genome ID
**Subdivision 1**							
Terriglobus roseus KBS 63[3]	this study	grassland soil[1]	KBS 63	CP003379.1	3572	GCA_000265425.1	2510461070
Terriglobus sp. TAA 43	this study	termite hindgut[1]	TAA 43	JUGR00000000.1	18239	GCA_000800015.1	2585428133
Acidobacteriaceae bacterium KBS 89	this study	grassland soil[1]	KBS 89	ARME00000000.1	30243	GCA_000381605.1	2522125061
Acidobacteriaceae bacterium KBS 146	this study	grassland soil[1]	KBS 146	JHVA00000000.1	31960	GCA_000688615.1	2556793019
Acidobacteriaceae bacterium TAA 166	this study	termite hindgut[1]	TAA 166	ATWD00000000.1	30011	GCA_000421065.1	2524614882
Acidobacteriaceae bacterium KBS 83	this study	agricultural soil[1]	KBS 83	ARMD00000000.1	30242	GCA_000381585.1	2519899651
Acidobacterium capsulatum ATCC51196[3]	(1)	acid mine drainage[2]	Acap	CP001472.1	1086	GCA_000022565.1	643692001
Granulicella tundricola MP5ACTX9[3]	(2, 3)	tundra soil[1]	Grtund	CP002480.1	13764	GCA_000178975.2	649633002
Granulicella mallensis MP5ACTX8[3]	(2, 3)	tundra soil[1]	Grmall	CP003130.1	2833	GCA_000178955.2	2503982037
Acidobacterium ailauui PMMR2	(4)	geothermally-heated Hawaiian microbial mat[2]	Aaila	JIAL00000000.1	13755	GCA_000688455.1	2558860986
"*Candidatus* Koribacter versatilis" Ellin345[3]	(1)	pasture soil[1]	Ellin 345	CP000360	1384	GCA_000014005.1	2606217699
Edaphobacter aggregans DSM19664	With permission KMG-I project, (N.Kyrpides)	forest soil[1]	Eaggr	JQKI00000000.1	33197	GCA_000745965.1	2571042012
Silvibacterium bohemicum S15	(5)	coniferous soil[1]	Silvib	LBHJ00000000.1	38174	GCA_001006305.1	2654587641

Terracidiphilus gabretensis S55	(6)	forest soil[1]	Tgab	LAIJ00000000.1	41667	GCA_001449115.1	2648501865
Terriglobus saanensis SP1PR4[3]	(3, 7)	tundra soil[1]	Tsaan	CP002467.1	2908	GCA_000179915.2	649633100
**Subdivision 3**							
Acidobacteria bacterium KBS 96	this study	agricultural soil[1]	KBS 96	ARMF00000000.1	30244	GCA_000381625.1	2522125054
Bryobacter aggregatus MPL3	this study	acidic Sphagnum peat bog[1]	Bryagg	JNIF00000000.1	32097	GCA_000702445.1	2562617048
"Candidatus Solibacter usitatus" Ellin6076[3]	(1)	pasture soil[1]	Ellin 6076	CP000473	1167	GCA_000014905.1	639633060
**Subdivision 4**							
Chloracidobacterium thermophilum B[3]	(8)	alkaline microbial mats[2]	Chlortherm	CP002514.1, CP002515.1	10823	GCA_000226295.1	2512047033
Pyrinomonas methylaliphatogenes K22	(9)	geothermal soil[2]	Pyrmet	CBXV000000000	35807	GCA_000820845.2	2645727641, 2545824503
**Subdivision 6**							
Luteitalea pratensis DSM100886[3]	(10)	German soil[1]	Lprat	CP015136	44495	GCA_001618865.1)	2687453111
**Subdivision 8**							
Geothrix fermentans DSM14018	With permission KMG-I project, (N.Kyrpides)	hydrocarbon-contaminated aquifer[2]	Gferm	NZ_AUAU00000000	11064	GCA_000428885.1	2523231062
Holophaga foetida TMBS4	(11)	anoxic freshwater mud[2]	Hfoet	AGSB00000000	10811	GCA_000242615.3	2509601028
**Subdivision 23**							
Thermoanaerobaculum aquaticum MP-01	(12)	freshwater hot spring[2]	Thermaq	JMFG00000000	31925	GCA_000687145.1	2579778550

[1]These genomes were defined as 'soil' environments stemming from temperate (grassland, agricultural, peat, coniferous and forest soils) and Arctic soils. They are referred to in the text as 'soil'. The 'termite associated acidobacteria' were placed in this category as preliminary data suggests that are not autochthonous to the guts, rather zymogenous inhabitants of the gut (13).

[2]These genomes were defined as 'non temperate or Arctic soil' environments stemming from anoxic mud, hot spring, contaminated aquifer, geothermal soil, alkaline or thermophilic microbial mats, and acid mind drainage referred to in the text as "other".

[3]Indicates a closed genome.

References:

1. Ward N.J., Challacombe J.F., Janssen P.H., Henrissat B., Coutinho P.M., Wu M., *et al.* (2009) Three genomes in the phylum *Acidobacteria* provide insight into their lifestyles in soils. *Appl Environ Microbiol* **74**:2046–2056.

2. Rawat S.R., Mannisto M.K., Starovoytov V., Goodwin L., Nolan M., Hauser L., *et al*. (2014) Complete genome sequence of *Granulicella tundricola* type strain MP5ACTX9(T), an *Acidobacteria* from tundra soil. *Stand Genomic Sci* **9**:449–461.

3. Rawat S.R., Mannisto M.K., Bromberg Y., Haggblom M.M. (2012) Comparative genomic and physiological analysis provides insights into the role of *Acidobacteria* in organic carbon utilization in Arctic tundra soils. *FEMS Microbiol Ecol* **82**:341–355.

4. Myers M.R. and King G.M. (2016) Isolation and characterization of *Acidobacterium ailaaui* sp. nov., a novel member of *Acidobacteria* sub-division I, from a geothermally-heated Hawaiian microbial mat. *Int J Syst Evol Microbiol* **66**: 5328-5335

5. Lladó S., Benada O., Cajthaml T., Baldrian P., García-Fraile P. (2016) *Silvibacterium bohemicum* gen. nov. sp. nov., an acidobacterium isolated from coniferous soil in the Bohemian Forest National Park. *Syst Appl Microbiol* **39**:14–19.

6. García-Fraile P., Benada O., Cajthaml T. (2016) *Terracidiphilus gabretensis* gen. nov., sp. nov., an abundant and active forest soil acidobacterium important in organic matter transformation. *Appl Env MIcrobiol* **82**:560-569.

7. Rawat S.R., Mannisto M.K., Starovoytov V., Goodwin L., Nolan M., Hauser L., *et al*. (2012) Complete genome sequence of *Terriglobus saanensis* type strain SP1PR4(T), an *Acidobacteria* from tundra soil. Stand Genomic Sci 7:59–69.

8. Garcia Costas A.M., Liu Z., Tomsho L.P., Schuster S.C., Ward D.M., Bryant D.A. (2012) Complete genome of Candidatus *Chloracidobacterium thermophilum*, a chlorophyll-based photoheterotroph belonging to the phylum *Acidobacteria*. *Environ Microbiol* **14**:177–190.

9. Greening C., Carere C.R., Rushton-Green R., Harold L.K., Hards K., Taylor M.C., *et al*. (2015) Persistence of the dominant soil phylum *Acidobacteria* by trace gas scavenging. *Proc Natl Acad Sci USA* **112**:10497–10502.

10. Huang S., Vieira S., Bunk B., Riedel T., Spröer C., Overmann J. (2016) First complete genome sequence of a subdivision 6 acidobacterium strain. *Genome Announ* **4**:e00469–16.

11. Anderson I., Held B., Lapidus A., Nolan M., Lucas S., Tice H., *et al*. (2012) Genome sequence of the homoacetogenic bacterium *Holophaga foetida* type strain (TMBS4(T)). *Stand Genomic Sci* **6**:174–184.

12. Stamps B.W., Losey N.A., Lawson P.A., Stevenson B.S. (2014) Genome sequence of *Thermoanaerobaculum aquaticum* MP-01T, the first cultivated member of *Acidobacteria* subdivision 23, isolated from a hot spring. *Genome Announ* **2**:e00570–14–e00570–14.

13. Stevenson B.S., Eichorst S.A., Wertz J.T., Schmidt T.M., Breznak J.A. (2004) New strategies for cultivation and detection of previously uncultured microbes. *Appl Environ Microbiol* **70**:4748–4755.

Table S2. General genome features across new genomes from this study.

	Terriglobus roseus	*Terriglobus* sp.	*Acidobacteriaceae bacterium*	*Acidobacteriaceae bacterium*	*Acidobacteriaceae bacterium*	*Acidobacteriaceae bacterium*	*Bryobacter aggregantus*	*Acidobacteria bacterium*
Strain	KBS 63	TAA 43	KBS 89	KBS 83	TAA 166	KBS 146	MPL3	KBS 96
Subdivision	1	1	1	1	1	1	3	3
Genome data								
Genome size (bp)	5 227 858	4 947 033	6 009 926	6 250 814	6 136 933	5 001 037	5 747 993	6 692 160
DNA coding region (bp)	4 688 148	4 525 334	5 322 921	5 470 371	5 398 159	4 489 703	5 399 614	6 053 597
G+C content (mol%)	60	57	58	59	59	57	58	57
Total number of genes	4399	4262	5119	5491	5364	4230	5126	5779
Pseudogenes	92	37	110	0	254	88	144	240
RNA genes								
tRNA genes	53	47	49	47	48	49	51	71
rRNA genes	6	3	3	3	3	3	6	6
No. rRNA operons	2	1	1	1	1	1	2	2
Other RNA genes	2	4	4	5	6	5	4	7
Total protein CDSs (%)								
With function prediction (%)	3220(73.2)	3141(73.7)	3605(70.42)	3755(68.38)	3837(71.53)	3209(75.86)	3684(71.87)	4193(71.52)
With COGs (%)	2557(58.13)	2465(57.84)	2802(54.74)	2841(51.74)	2975(55.46)	2586(61.13)	2781(54.25)	3214(54.82)
With Pfam domains	3,351(76.18)	3248(76.21)	3,785(73.94)	3,914(71.28)	3963(73.88)	3325(78.61)	3819(74.5)	4357(74.31)
Coding for signal peptides (%)	765(17.39)	808(18.96)	800(15.63)	744(13.55)	648(12.08)	670(15.84)	612(11.94)	718(12.25)
Coding for transmembrane proteins (%)	1097(24.94)	1034(24.26)	1344(26.26)	1420(25.86)	1233(22.99)	1059(25.04)	1043(20.35)	1364(23.26)

Table S3. Genome completeness, contamination and strain heterogeneity across all investigated genomes based on CheckM.

Species/Genome	Genome Assembly	Size (Mb)	GC (%)	Genes	Proteins	Completeness (%)	Contamination (%)	Strain Heterogeneity (%)
**Subdivision 1**								
Terriglobus roseus KBS 63*	GCA_000265425.1	5.23	60.3	4319	4176	100	13.79	94.44
Terriglobus sp. TAA 43	GCA_000800015.1	4.95	56.7	4178	4079	100	2.59	0
Acidobacteriaceae bacterium KBS 89	GCA_000381605.1	6.01	57.6	4992	4835	100	0.86	0
Acidobacteriaceae bacterium KBS 146	GCA_000688615.1	5.00	56.7	4124	3989	100	0	0
Acidobacteriaceae bacterium TAA 166	GCA_000421065.1	6.14	58.8	5161	4975	99.14	4.48	0
Acidobacteriaceae bacterium KBS 83	GCA_000381585.1	6.25	59.2	5273	5079	99.14	0	0
Acidobacterium capsulatum ATCC51196*	GCA_000022565.1	4.13	60.5	3396	3281	100	0.85	0
Granulicella tundricola MP5ACTX9*	GCA_000178975.2	5.50	59.9	4687	4542	99.15	0.85	0
Granulicella mallensis MP5ACTX8*	GCA_000178955.2	6.24	57.9	4844	4735	99.14	2.63	0
Acidobacterium ailaaui PMMR2	GCA_000688455.1	3.69	56.5	3159	3043	100	0	0
"*Candidatus* Koribacter versatilis" Ellin345*	GCA_000014005.1	5.65	58.4	4972	4860	99.95	0	0
Edaphobacter aggregans DSM19664	GCA_000745965.1	8.18	58.7	7121	6610	100	1.72	0
Silvibacterium bohemicum S15	GCA_001006305.1	6.46	58.2	5292	5117	100	3.85	0
Terracidiphilus gabretensis S55	GCA_001449115.1	5.35	57.3	4386	4244	99.78	0.86	0
Terriglobus saanensis SP1PR4*	GCA_000179915.2	5.10	57.3	4261	4163	100	1.72	0
**Subdivision 3**								
Acidobacteria bacterium KBS 96	GCA_000381625.1	6.69	57.2	5746	5567	97.39	0.87	0
Bryobacter aggregatus MPL3	GCA_000702445.1	5.75	57.9	5051	4918	98.26	1.74	0
"*Candidatus* Solibacter usitatus" Ellin6076*	GCA_000014905.1	9.97	61.9	8113	7991	100	0.88	0
**Subdivision 4**								
Chloracidobacterium thermophilum B*	GCA_000226295.1	3.69	61.3	3034	2905	95.71	2.56	0
Pyrinomonas methylaliphatogenes K22	GCA_000820845.2	3.79	59.4	3216	3067	96.53	1.71	0
**Subdivision 6**								
Luteitalea pratensis DSM100886*	GCA_001618865.1	7.48	67.2	6293	6200	97.44	5.13	16.67
**Subdivision 8**								
Geothrix fermentans DSM 14018	GCA_000428885.1	3.29	68.9	2925	2806	99.12	0	0
Holophaga foetida TMBS4	GCA_000242615.3	4.13	62.9	3581	3471	99.12	0.88	0
**Subdivision 23**								
Thermoanaerobaculum aquaticum MP-01	GCA_000687145.1	2.66	63.0	2393	2251	94.44	1.71	0

*denotes a closed genome.

Table S4. Preliminary assessment of putative insertion sequence (IS) element families across the acidobacterial genomes. Abbreviations for the genomes can be found in Table S1. Numbers represent complete/partial/pseudogene/unknown IS elements. Analysis was performed using the ISsaga – IS Semi-automatic genomic annotation website (http://issaga.biotoul.fr/issaga_login.php?type=2). Putative IS elements need to be manually curated.

IS element families	Acap	A.ailaaui	Gtund	Gmall	Ellin345	TAA43	Tsaan	KBS63	KBS146	Edapho	TAA166	KBS83	KBS89	Silvib	Tgab	Ellin6076	KBS96	MPL3	Chloroacd	K22	Lprat	Hfoet	Gferm	Thermana
IS1182				0,0,0,1		1,0,0,0	0,1,0,0			0,4,0,2	3,0,0,0	0,2,0,0			0,0,0,1	0,0,0,1	0,1,0,0				0,1,0,7			
ISNCY_ssgr_IS1202									0,1,0,0	3,14,0,5				1,0,0,0		0,0,0,4	2,0,0,0							0,1,0,1
IS66			0,1,0,1				0,2,0,1			2,2,0,6				2,4,0,2		1,0,0,1	0,3,0,0	2,1,0,2						
IS701										0,6,0,6						1,0,0,9								
IS630										4,13,0,10					1,0,0,0	1,1,0,6		10,5,0,14			3,7,0,1			
ISL3	0,0,0,2	0,0,0,2	2,1,0,1	0,0,0,2	0,0,0,2	0,0,0,1	0,2,0,5	0,2,0,7	0,1,0,8	0,4,0,7	1,0,0,0	0,2,0,2			0,0,0,3	1,4,0,2	0,0,0,4	0,1,0,2	0,0,0,2	0,1,0,3		1,1,0,5	0,5,0,1	
IS481	11,1,0,1		1,0,0,0	1,0,0,2					0,1,0,1	1,5,0,3	0,3,0,0	0,0,0,1		0,2,0,0	1,0,0,1	12,0,0,1		9,3,0,1		0,0,0,1	4,0,0,0		0,15,0,0	0,1,0,0
IS3_ssgr_IS3	0,0,0,1									1,2,0,0						15,0,0,14								
IS110	2,0,0,0	1,2,0,1		2,4,0,0						2,19,0,15			0,5,0,0	1,0,0,0	0,2,0,0	18,0,0,3	1,3,0,5	0,0,0,1			1,0,0,3		0,1,0,1	
IS6										3,6,0,2	0,1,0,0	0,2,0,1				3,4,0,0								
IS91										6,13,0,12						4,1,0,6	0,2,0,0					3,0,0,9		
IS21	3,3,0,3	1,0,0,1								2,7,0,3						5,1,0,3	3,6,0,2				7,2,0,3	8,0,0,3		
IS3_ssgr_IS407	5,2,0,0	4,2,0,0	0,3,0,0	0,1,0,1			0,1,0,0			5,12,0,2	21,12,0,2			1,9,0,3	2,4,0,0		0,0,0,2	2,0,0,0						
IS5				4,0,0,0						0,2,0,0				0,0,0,1			0,1,0,0							
IS3_ssgr_IS3										0,1,0,1							0,1,0,0				1,1,0,0			
IS630		0,1,0,0								0,5,0,4							0,1,0,2			0,0,0,2		0,0,0,1		
IS110_ssgr_IS1111		1,0,0,2	7,0,0,0	1,0,0,1	5,0,0,2		3,2,0,0	0,4,0,0		2,21,0,5	3,2,0,0	0,2,0,6		0,1,0,1			3,0,0,1				0,0,0,3			

**IS element families**	**Acap**	**A.ailaaui**	**Gtund**	**Gmall**	**Ellin345**	**TAA43**	**Tsaan**	**KBS63**	**KBS146**	**Edapho**	**TAA166**	**KBS83**	**KBS89**	**Silvib**	**Tgab**	**Ellin6076**	**KBS96**	**MPL3**	**Chloroacd**	**K22**	**Lprat**	**Hfoet**	**Gferm**	**Thermana**
IS256		0,1,0,5															5,0,0,2			0,12,0,0	3,0,0,0			
IS4_ssgr_IS4Sa										0,3,0,1								0,2,0,0						
IS3_ssgr_IS51																		0,3,0,0						
IS5_ssgr_IS1031										3,8,0,4								0,6,0,13						
IS5_ssgr_IS427	3,0,0,0									3,2,0,3	1,2,0,0	2,4,0,5		3,5,0,1				4,10,0,12				1,0,0,1		
IS5_ssgr_IS5	1,2,0,0	0,3,0,0	16,4,0,0							2,17,0,0		0,2,0,0	0,1,0,0					9,0,0,0						
ISNCY_ssgr_ISPlu15																			0,0,0,2					
IS200_IS605		2,1,0,0																	0,1,0,1	2,0,0,1				
IS200_IS605_ssgr_IS1341																			0,2,0,1	0,0,0,10				
IS607																			3,1,0,0	2,0,0,0				
ISAs1										0,3,0,3	1,0,0,0									0,0,0,1		6,0,0,0		
IS1595_ssgr_ISPna2																				0,0,0,1				
IS66_ssgr_ISBst12																						0,0,0,12		
IS1380										0,1,0,14												1,0,0,0		
ISNCY_ssg_ISLbi1																						1,0,0,0		0,2,0,0
IS1634																						1,2,0,2		
IS200_IS605_ssgr_IS200				0,0,0,1		0,0,0,2		0,0,0,2		1,0,0,0												2,2,0,0		
IS1595_ssgr_IS1595										0,0,0,1												5,1,0,1		
IsoAzo_13										0,0,0,4													0,0,0,2	0,0,0,1

**IS element families**	**Acap**	**A.ailaaui**	**Gtund**	**Gmall**	**Ellin345**	**TAA43**	**Tsaan**	**KBS63**	**KBS146**	**Edapho**	**TAA166**	**KBS83**	**KBS89**	**Silvib**	**Tgab**	**Ellin6076**	**KBS96**	**MPL3**	**Chloroacd**	**K22**	**Lprat**	**Hfoet**	**Gferm**	**Thermana**
ISNCY	1,0,0,0		1,0,0,0	1,0,0,0	1,0,0,1	1,0,0,0	2,0,0,0	1,0,0,0				2,0,0,0	3,0,0,0	2,0,0,0	1,0,0,0								0,0,0,5	
ISNCY_ssgr_IS892																							0,0,0,9	
IS1595_ssgr_ISNwi1	0,0,0,1		0,0,0,2	0,1,0,2					0,0,0,1						0,0,0,2									
IS4_ssgr_IS50			0,1,0,2																					
IS30			1,0,0,0	8,0,0,0			6,0,0,0				3,0,0,0													
IS1_ssgr_ISMhu11															1,0,0,0									
IS1595_ssgr_ISSod11										0,6,0,0														
IS3_ssgr_IS150																					0,3,0,2			
IS5_ssgr_IS903																					2,10,0,22			
Tn3										2,2,0,4														

Table S5. Locus tag for the genes identified across the acidobacterial genomes encoding for the catalytic subunit of respiratory oxygen reductases (cytochrome terminal oxidases). Locus tags for each gene starts with the ID given in column "locus tag start", followed by the number in the respective column.

**Genome**	**Locus tag**			
	**Locus tag start**	**HCO[1] type A**	**HCO[1] type C**	***bd* cytochrome oxidase**
"*Candidatus* Koribacter versatilis" Ellin345	Acid345_	2995<br>0438	ND	3252
Terriglobus saanensis SP1PR4	AciPR4_	0757<br>1977<br>1384<br>4039<br>1735	ND	ND
Terriglobus roseus KBS 63	Terro_	0292<br>4044	4248	ND
Terriglobus sp. TAA 43	M504DRAFT_	1755<br>2704<br>1876	ND	ND
Granulicella mallensis MP5ACTX8	AciX8_	1358<br>0184<br>2887	ND	2831
Granulicella tundricola MP5ACTX9	AciX9_	0075<br>1502<br>2163	ND	ND
Acidobacteriaceae bacterium KBS 89	G003DRAFT_	1427<br>1559<br>2097	ND	ND
Acidobacteriaceae bacterium KBS 146	M015DRAFT_	2746	ND	0063
Acidobacteriaceae bacterium TAA 166	H979DRAFT_	2562<br>4938	ND	5221<br>0828 (fragmented)
Edaphobacter aggregans DSM 19364	Q363DRAFT_	00800<br>00183<br>03851<br>06175<br>01386	03854<br>03822	01553<br>04806 (only 113 aa)
Terracidiphilus gabretensis S55	Ga0102063_	1091461<br>109341	ND	ND
Acidobacteriaceae bacterium KBS 83	G002DRAFT_	03417<br>03882<br>04166<br>04897	ND	ND
Acidobacterium capsulatum ATCC 51196	ACP_	2437	0885	0468

Genome/Strain	Locus tag			
	**Locus tag start**	**HCO[1] type A**	**HCO[1] type C**	***bd* cytochrome oxidase**
Silvibacterium bohemicum S15	Ga0077217_	10214<br>10884<br>102165<br>103529	ND	103203<br>103592
Acidobacterium ailaaui PMMR2	N655DRAFT_	2211<br>1168<br>0997	1334	0369<br>1326
"Candidatus Solibacter usitatus" Ellin6076	Acid_	5590<br>0498<br>7581<br>6908	3507	6681
Acidobacteria bacterium KBS 96	G004DRAFT_	1132<br>1673<br>5485<br>4385	0635	ND
Bryobacter aggregatus MPL3	M017DRAFT_	4951<br>4036	ND	ND
Chloracidobacterium thermophilum B	Cabther_	B0233	ND	A0912
Pyrinomonas methylaliphatogenes K22	Ga0098325_	108284	ND	10780
Luteitalea pratensis DSM100886	Ga0133452_	11386<br>115325	114699	115305
Holophaga foetida DSM 6591	HolfoDRAFT_	0900	ND	1360
Geothrix fermentans DSM 14018	G398DRAFT_	02716	ND	02386<br>00741
Thermoanaerobaculum aquaticum MP-01	EG19_	02365	ND	10715

[1]HCO=heme-copper oxygen reductase/terminal oxidases, ND=not detected

Table S6. Marker genes identified for nitrogen metabolism across acidobacterial genomes. Columns provide the function ID (EC or TC number), product (enzyme, transporter) name, gene name and locus numbers for each genome represented by its strain name.

			SD 1															SD 3			SD 4		SD 6	SD 8		SD 23
**Function ID**	**Product**	**Gene name**	**Ellin 345<br>Acid345_**	**SP1PR4<br>AciPR4_**	**KBS 63<br>Terro_**	**TAA 43<br>M504DRAFT_**	**MP5ACTX8<br>AciX8_**	**MP5ACTX9<br>AciX9_**	**KBS 89<br>G003DRAFT**	**KBS 146<br>M015DRAFT_**	**TAA 166<br>H979DRAFT_**	**DSM19364<br>Q363DRAFT_**	**S55<br>Ga0102063_**	**KBS 83<br>G002DRAFT_**	**ATCC51196<br>ACP_**	**S15<br>Ga0077217_**	**PMMR2<br>N655DRAFT_**	**Ellin6076<br>Acid_**	**KBS 96<br>G004DRAFT_**	**MPL3<br>M017DRAFT_**	**B<br>Cabther_**	**K22<br>Ga0098325_**	**DSM100886<br>Ga0133452_**	**TMBS4<br>HolfoDRAFT_**	**DSM14018<br>G398DRAFT_**	**MP-01<br>EG19_**
**Dissimilatory nitrate and nitrite reduction (denitrification)**																										
EC:1.7.99.4	Membrane-bound nitrate reductase	*narG* operon	ND	ND	ND	ND	ND	ND	ND	ND	ND	ND	ND	ND	ND	ND	ND	ND	ND	ND	ND	ND	ND	ND	01357-01360	ND
EC:1.7.99.4	Periplasmic nitrate reductase	*napA* operon	ND	ND	ND	ND	ND	ND	ND	ND	ND	ND	ND	ND	ND	ND	ND	ND	ND	ND	ND	ND	ND	ND	ND	12345-12365
EC:1.7.2.1	Copper-containing nitrite reductase	*nirK*	ND	ND	ND	ND	ND	ND	ND	ND	ND	ND	ND	ND	ND	ND	ND	ND	3028<br>3603	ND	ND	ND	ND	ND	ND	ND
EC:1.7.2.2	Nitrite reductase (cytochrome; ammonia froming)	*nrfHA*	ND	ND	ND	ND	ND	ND	ND	ND	ND	ND	ND	ND	ND	ND	ND	ND	ND	ND	ND	ND	115645<br>115644	1771/1769<br>1772/1770	00889<br>00888	ND
EC:1.7.2.5	Nitric-oxide reductase	*norBC/norZ*	0364	ND	0243	ND	ND	ND	ND	ND	103454	01282<br>04947	ND	ND	ND	103454	0246	7864<br>2936	ND	ND	ND	ND	112424<br>112423	ND	00261<br>00671	05590<br>05595
EC:1.7.2.4	Nitrous-oxide reductase	*nosZ*	ND	ND	ND	ND	ND	ND	ND	ND	ND	ND	ND	ND	ND	ND	ND	ND	ND	ND	ND	ND	112005	ND	ND	ND
**Assimilatory nitrate and nitrite reduction**																										
EC:1.7.99.4	Nitrate reductase	*nasA*	ND	ND	1211	3864	0895	0125	2174	0852	ND	05846	ND	ND	ND	ND	3124	0183<br>2928	0571<br>3063	ND	ND	10117<br>10441	111517<br>11132	ND	01348	ND
EC:1.7.1.15	Nitrite reductase (NADH)	*nirB*	ND	ND	ND	ND	ND	ND	ND	ND	ND	ND	ND	ND	ND	ND	ND	0184	0570	ND	ND	10115	111518	ND	ND	ND
EC:1.7.7.1	Ferredoxin-nitrite reductase	*nirA*	ND	ND	1214	3867	0892	0128	2177	0855	ND	ND	ND	04105<br>04104	ND	ND	3127	ND	ND	ND	ND	107237	ND	ND	ND	ND
TC 2.A.1.8	Nitrate/ nitrite porter (NNP)	*narK/ NRT*	ND	ND	1210	3863	0896	0124	2173	0851	0043<br>0694<br>3072	05844	ND	595	ND	ND	3123	0186<br>2929	3064	4414	ND	10118<br>10445	111520<br>11133	ND	ND	ND
**Ammonia assimilation**																										
EC:6.3.1.2	Glutamine synthetase (GS)	*glnA*	3142<br>0166	1794	1807	0692	1285	1375	3064	4032	3823	02162	11234	01403<br>04563	1250<br>0595	104762	2561	4831<br>1901<br>0067	2781<br>4790	1794<br>1790	A2003	104218	116176<br>113561	0125	01680	09235
EC:1.4.1.-13/14	Glutamate synthase (GOGAT), large chain	*gltB*	3680	2050	1714	0089	2097	1897	2498	0072	4010	01545	107121	03977	2997	119172	3103	3816	5814	0322	A1316	108822	115550	0249	00368	04200
EC:1.4.1.-13/14	Glutamate synthase (GOGAT), small chain	*gltD*	2796<br>3679	ND	4344	ND	2687	0264	2908	ND	ND	04267	110352	ND	1387	102335	ND	3815<br>0543<br>3510<br>7661	5815	0323	ND	108823<br>108709<br>113556	115549<br>114103	0248<br>2650<br>0142<br>3463<br>2931	01329	08420<br>02560
EC:1.4.1.2	Glutamate dehydrogenase (NAD) (GDH)	*gdh2*	ND	ND	ND	ND	ND	ND	ND	ND	ND	ND	ND	ND	ND	ND	ND	ND	ND	ND	ND	ND	115662	ND	ND	ND
EC:1.4.1.-3/4	Glutamate dehydrogenase (NAD(P)(+)/NADP(+)) (GDH)	*gdhA*	0943<br>2942<br>4115	1275	2787	0923	0803	3035	3262	3517	1094	05027	11259	02399	2883	102135	2044	0039<br>4761	0737<br>4563	0363<br>1312	A2079<br>B0781	10526	ND	1742<br>2764	01097<br>01591<br>02884	08250
**Ammonium uptake transport**																										
TC 1.A.11	Ammonium transporter	*amtB*	1490<br>3596<br>4446	0992	1497	0950	4320<br>3327	2274	2799	4215	0572	02736	1064	03980	1255<br>1252<br>0919	101403	0634	7190<br>5532	0661<br>3003	1792	A0160	108593	113472<br>113167	0124<br>0122<br>3429	00569<br>00857	ND
-	Nitrogen regulatory protein P-II	*glnK*	1489<br>4445	0991	1496	0951	4321<br>3326	2273	2798	4216	0571	02735	1065	03979	0920	101402	0635	7189<br>5534	0660	1793	A0161	108595	113471	0123<br>0121<br>3428	00568<br>00856	ND

Table S6. Marker genes identified for nitrogen metabolism across acidobacterial genomes. Columns provide the function ID (EC or TC number), product (enzyme, transporter) name, gene name and locus numbers for each genome represented by its strain name.

			SD 1															SD 3			SD 4		SD 6	SD 8		SD 23
**Function ID**	**Product**	**Gene name**	**Ellin 345<br>Acid345_**	**SP1PR4<br>AciPR4_**	**KBS 63<br>Terro_**	**TAA 43<br>M504DRAFT_**	**MP5ACTX8<br>AciX8_**	**MP5ACTX9<br>AciX9_**	**KBS 89<br>G003DRAFT**	**KBS 146<br>M015DRAFT_**	**TAA 166<br>H979DRAFT_**	**DSM19364<br>Q363DRAFT_**	**S55<br>Ga0102063_**	**KBS 83<br>G002DRAFT_**	**ATCC51196<br>ACP_**	**S15<br>Ga0077217_**	**PMMR2<br>N655DRAFT_**	**Ellin6076<br>Acid_**	**KBS 96<br>G004DRAFT_**	**MPL3<br>M017DRAFT_**	**B<br>Cabther_**	**K22<br>Ga0098325_**	**DSM100886<br>Ga0133452_**	**TMBS4<br>HolfoDRAFT_**	**DSM14018<br>G398DRAFT_**	**MP-01<br>EG19_**
**Amino acid (AA) transport**																										
TC 2.A.3	AA-polyamine-organocation (APC) superfamily	-	3835<br>3036<br>0162<br>0976<br>0678<br>1536<br>1679<br>3333<br>0046<br>3285<br>2701<br>3016<br>3114<br>1474<br>3284<br>2156<br>3351	1416<br>2037<br>3497<br>2464<br>0020<br>2651<br>2036<br>0364<br>3374<br>0178<br>3964	4224<br>3913<br>0836<br>3040<br>2261<br>3794<br>1607<br>1744<br>4002<br>1745<br>1504<br>2625	0571<br>0973<br>4186<br>1421<br>0465<br>2262<br>1033<br>0974	2786<br>1117<br>1565<br>1566<br>4112<br>2732<br>0955<br>4779<br>2758<br>0411<br>4050<br>2078<br>2255	3472<br>1886<br>2694<br>3345<br>0983<br>2124<br>1885<br>3541<br>1735	2480<br>3866<br>1151<br>3528<br>2967<br>4692<br>2481<br>3361<br>0152<br>2979<br>4815<br>2165	0789<br>0094<br>0088<br>1963<br>3716<br>0087<br>3264<br>0803<br>0429	4308<br>4024<br>3310<br>3131<br>4700<br>4844<br>4422<br>4025<br>4714<br>2121	02969<br>04137<br>06911<br>01532<br>02982<br>02841<br>01525<br>00909<br>06534<br>01136<br>01531<br>04348	101130<br>112108<br>110422<br>1091158<br>1091361<br>102161<br>101213<br>101180<br>109238<br>102162<br>102153<br>10425<br>1015	04930<br>00444<br>01650<br>02984<br>04564<br>04463<br>02605<br>02850<br>03907<br>01718<br>02065<br>04646<br>04042	3401<br>1795<br>0121<br>1264<br>0122<br>3520<br>0855<br>1500<br>3151	103118<br>104794<br>11833<br>104291<br>119163<br>11415<br>104624<br>103418<br>104282<br>11416<br>119135<br>120582<br>112218<br>104387<br>103119<br>104110<br>104225	0731<br>2168<br>2616<br>1848<br>1530<br>1461<br>2511<br>2510<br>0180<br>2944	7162<br>2823<br>2999<br>0814<br>3825<br>4914<br>5796<br>4943<br>2124<br>1822<br>1175<br>1927<br>4944<br>4619<br>2296<br>1835<br>1245<br>4783	2943<br>0110<br>4012<br>3303<br>2272<br>4788<br>1038<br>0248<br>0923<br>3740<br>2626<br>4366	3654<br>3019<br>3778<br>4169<br>3168<br>2459<br>3793<br>4168<br>2862	A0283<br>A1466<br>B0209	108537<br>104442<br>108342<br>10549<br>10713<br>108598<br>10550<br>108850<br>10639<br>101308	112573<br>111053<br>113432<br>115635<br>115468<br>114739<br>11630<br>114707<br>115073<br>112092<br>114166	2468<br>0909<br>2467	00939<br>00553<br>01205<br>01204<br>02692	03995<br>08400<br>07245
TC 2.A.23	Dicarboxylate/AA:cation symporter (DAACS) family	-	2088	1150	3462	1300	1251<br>1919	0511<br>2737	0046<br>4779	3790	0690	00653	109422	00209	ND	104275	2012	3691<br>1117	ND	3990	B0019	107111	112162<br>114989	ND	01633<br>01569	04235
TC 3.A.1.4.-	Branched chain hydrophobic AA (HAAT) transporter (ABC superfamily)	*livKFGHM*	ND	ND	ND	ND	ND	ND	ND	ND	ND	ND	ND	ND	ND	ND	ND	6083	ND	ND	A0310<br>A0414<br>A0416<br>B0208<br>B0693	ND	ND	0334-0338<br>0471-0475<br>1865-1869<br>0012<br>0020<br>0049<br>0434<br>1336<br>1660<br>2091<br>3541	00619-00623<br>01283-01287<br>02586-02589<br>02014	00910<br>10105<br>10165<br>10890
**Nitrogen fixation**																										
EC 1.18.6.1	Nitrogenase	*nifH* operon	ND	ND	ND	ND	ND	ND	ND	ND	ND	ND	ND	ND	ND	ND	ND	ND	ND	ND	ND	ND	ND	0884-0892	ND	ND
**Proteases: select peptidases**																										
EC:3.4.21.-	Serine endopeptidases family S8 (subtilisin-like) and family S53	-	2635<br>3141<br>4222	1724	1995<br>2361<br>2960<br>3758	0214<br>1078<br>1356<br>3807	0674<br>3104	0612<br>1366<br>2897	1754<br>4086<br>4513	0225<br>3702	3468<br>3298<br>5203	02302<br>03219<br>04118	101114<br>1091596	01357<br>03150	1810	10438<br>101563<br>114128	1743	7045<br>6709<br>5611<br>3452<br>6185<br>0578<br>3135	3271<br>1283<br>5333<br>2600	3675<br>1502<br>3514<br>2644<br>4202	A0104<br>A0163<br>A0211<br>A1826<br>A1515<br>B0297	108125<br>108305<br>105147<br>106189<br>102293<br>108463	112415<br>116070<br>113401<br>111417<br>114755<br>111450<br>115742	ND	ND	04460<br>09280
EC:3.4.24.-	Metallopeptidase family M3	-	1180	4110	1333	1014	3979<br>4246	2083	4538	2775	2381	00038	112387	02390	2228	11292<br>102128	2060	7278<br>5816	2784	1610	ND	ND	113228<br>113591	1677<br>3635	01094	01275
EC:3.4.24.-	Metalloendopeptidase family M4	-	ND	ND	ND	ND	ND	ND	ND	ND	ND	ND	ND	ND	ND	ND	ND	ND	ND	ND	ND	ND	1179<br>111548	ND	00921<br>01202<br>01974	01045<br>11630<br>09070<br>09065
EC:3.4.24.-	Metallopeptidase family M13	-	1078<br>4516<br>4515<br>1491<br>0009	0730<br>2272	3760<br>4203<br>3972	1358<br>2791<br>2824<br>1950	3677<br>4565<br>0257<br>1217	4474<br>0774<br>0014<br>2977	0313<br>3212<br>3722<br>4830<br>0755	3575<br>1401<br>1755	5263<br>1241<br>1022	01998<br>02505<br>01905<br>00004<br>00734	10728<br>109506	00149<br>00270<br>00269	1917<br>2297<br>2953	101692<br>104479	2759	2743<br>1534<br>2789<br>1818<br>4723	2785	3320	ND	ND	115371<br>113880	ND	01908<br>01909<br>02780	ND
EC:3.4.24.-	Metallopeptidase family M16	-	0622<br>1568<br>0621<br>4406	0158<br>1386	0079<br>1422	0170<br>2147	1363<br>1743<br>1364<br>0386	1564<br>2145<br>2146	2647<br>4241<br>4242<br>0644	4068<br>4069<br>0393	4632<br>4451<br>4450<br>1035	01422<br>01747<br>01746<br>03540	1091689<br>1091688<br>110289<br>10238<br>10810	03532<br>03533<br>02436<br>02075	0056<br>0482	101313<br>101314<br>101280	2801<br>2802<br>1801<br>1491	214-8/9<br>752-3/4<br>50-3/4<br>641-8/9<br>5508<br>1328<br>7201<br>2731<br>1734<br>5264	2456<br>2457<br>0912<br>0913<br>0429<br>0430<br>1695<br>2535	3433<br>3434<br>3153<br>3154<br>1857<br>0818	A0606<br>A0690<br>A1433<br>A0775<br>A1066<br>A1254<br>A0625<br>A1967	108712<br>108713<br>10312<br>10313<br>108415<br>108416<br>101318<br>102335<br>104338<br>108899	11572-1/2<br>1128-79/80<br>11488-0/1<br>1187-4/5<br>11867<br>111984<br>11536	2093<br>2493<br>1792	00902<br>00903<br>02791<br>00971	04450<br>04455<br>00340<br>11145

ND = not detected

Table S7. Locus tags across the acidobacterial genomes encoding for putative genes involved in fermentation pathways.

	Locus Tag						
**Genome**	L-lactate dehydrogenase (*ldh*, EC 1.1.1.27)	D-lactate dehydrogenase (*ldhA*,EC 1.1.1.28)	Formate C-acetyltransferase (*pflD*, EC 2.3.1.54)	Fumurate reductase/succinate dehydrogenase (*frdABC*/*sdhCAB*; EC 1.3.5.4/1.3.5.1)	Alchohol dehydrogenase (*adh*/*adhP*, EC 1.1.1.1)	Phosphate acetyltransferase/ Phosphate butyryltransferase (*pta*/*ptb*, EC 2.3.1.8/19)	Acetate kinase (*ackA*, EC 2.7.2.1)
**Subdivision 1**							
"Candidatus Koribacter versatilis" Ellin345 Acid345_	1483	ND	ND	138/3-5<br>257/0-2	0379<br>3044<br>4584	ND	ND
Terriglobus saanensis SP1PR4 AciPR4_	ND	ND	ND	101/0-2	1211	ND	0488
Terriglobus roseus KBS 63 Terro_	ND	ND	ND	107/2-4	2197<br>2561	ND	3746
Terriglobus sp. TAA 43 M504DRAFT_	ND	ND	ND	379/5-9	1157	ND	0424
Granulicella mallensis MP5ACTX8 AciX8_	ND	ND	ND	1299-1303	2812	ND	1456<br>4385
Granulicella tundricola MP5ACTX9 AciX9_	ND	ND	ND	138/6-9	2541<br>3151<br>4423	ND	0152<br>1690
Acidobacteriaceae bacterium KBS 89 G003DRAFT_	0265, 3782	ND	ND	30/46-50<br>43/48-50	1342<br>3131<br>4445	5071	3419<br>5070
Acidobacteriaceae bacterium KBS 146 M015DRAFT_	ND	ND	ND	4046<br>40/49-50	1075<br>2521<br>2893	3871	2059
Acidobacteriaceae bacterium TAA 166 H979DRAFT_	ND	ND	ND	3837<br>384/0-1	1955<br>2301<br>5366	ND	2034

Edaphobacter aggregans DSM19664 Q363DRAFT_	ND	ND	03979	0107/0-2<br>049/39-41<br>02176<br>021/79-81	00158<br>03835<br>05534<br>07321	03981	00865<br>03980<br>06211<br>06561
Terracidiphilus gabretensis S55 Ga0102063_	ND	ND	ND	10235/5-8	102323	107253	10232<br>10454
Acidobacteriaceae bacterium KBS 83 G002DRAFT_	ND	ND	ND	0190/3-5<br>0275/6-8<br>0417/7-9	02395<br>04049	ND	00108<br>03488
Acidobacterium capsulatum ATCC51196 ACP_	ND	ND	ND	010/1-3	0589<br>3493	0481	2233
Silvibacterium bohemicum S15 Ga0077217_	ND	ND	ND	10816/5-7	101678<br>103210<br>103237<br>120454	ND	10390<br>103263<br>104206
Acidobacterium ailaaui PMMR2 N655DRAFT_	ND	ND	ND	298/0-2	0373<br>2477	ND	0172
**Subdivision 3**							
"Candidatus Solibacter usitatus" Ellin6076 Acid_	ND	ND	ND	740/1-3	1558<br>5165<br>7928	1084<br>1330	3041<br>3255
Acidobacteria bacterium KBS 96 G004DRAFT_	ND	ND	ND	04/19-21	4761<br>5084<br>5508	ND	4429<br>5598
Bryobacter aggregatus MPL3 M017DRAFT_	ND	0131	ND	33/48-50	0776<br>4021<br>4026	ND	1118
**Subdivision 4**							
Chloracidobacterium thermophilum B Cabther_	A0304	ND	ND	A055/2-5	A1652<br>B0535	A1909	B0746
Pyrinomonas methylaliphatogenes K22 Ga0098325_	ND	ND	ND	10416/4-6	102184	108767	108839

**Subdivision 6**							
Luteitalea pratensis DSM100886 Ga0133452_	ND	111066	112212	1139/61-64 (frdABCD) 11415/2-4 1163/3-5	111091 112028 113099 115686	112214	112213
**Subdivision 8**							
Holophaga foetida TMBS4 HolfoDRAFT_	ND	ND	ND	045/6-8	3261	0402 1130	1418 3547
Geothrix fermentans DSM14018 G398DRAFT_	ND	01384	ND	0044/5-7	ND	00176 00396	00464
**Subdivision 23**							
Thermoanaerobaculum aquaticum MP-01 EG19_	ND	ND	ND	079/45-55	04475	06665	03040

ND = not detected

ND: EC 1.1.2.3; L-lactate dehydrogenase (cytochrome): *lldD*

Table S8. Locus tags for the genes identified across the acidobacterial genomes encoding for genes involved in heterotrophic carbon dioxide fixation. The complete locus tags for each genome are listed.

	**Locus Tag**				
**Genome**	Phosphoenolpyruvate carboxylase (EC:4.1.1.32)	Phosphoenolpyruvate carboxylase (EC:4.1.1.49)	Isocitrate dehydrogenase (EC:1.1.1.42)	2-ketoglutarate ferredoxin oxidoreductase (EC:1.2.7.3)	Pyruvate ferredoxin oxidoreductase (EC:1.2.7.1)
**Subdivision 1**					
"Candidatus Koribacter versatilis" Ellin345	Acid345_0537	ND	Acid345_1950	Acid345_0598, 0599, 0951, 0952, 1808, 1809, 1810, 1811	Acid345_0730
Terriglobus saanensis SP1PR4	AciPR4_0804	ND	AciPR4_1000	ND	ND
Terriglobus roseus KBS 63	Terro_4338	ND	Terro_0474,0475	ND	ND
Terriglobus sp. TAA 43	M504DRAFT_3277	ND	M504DRAFT_1564,1565	ND	ND
Granulicella mallensis MP5ACTX8	AciX8_0596	ND	AciX8_1286	ND	ND
Granulicella tundricola MP5ACTX9	AciX9_2691	ND		ND	ND
Acidobacteriaceae bacterium KBS 89	G003DRAFT4411	ND	G003DRAFT3061	G003DRAFT1319, 1320, 4283, 4284	ND
Acidobacteriaceae bacterium KBS 146	M015DRAFT_1423	ND	M015DRAFT_4036	ND	ND
Acidobacteriaceae bacterium TAA 166	H979DRAFT_4777	ND	H979DRAFT_3827	ND	ND
Edaphobacter aggregans DSM19664	Q363DRAFT_00717	ND	Q363DRAFT_02165	ND	ND
Terracidiphilus gabretensis S55	Ga0102063_1017	ND	Ga0102063_112310	Ga0102063_101309, 101310	Ga0102063_10929
Acidobacteriaceae bacterium KBS 83	ND	G002DRAFT_02415	G002DRAFT_02770	G002DRAFT_04297, 04298	ND
Acidobacterium capsulatum ATCC51196	ND	ACP_2868	ACP_0089,0990	ACP_0328, 0329	ACP_2686
Silvibacterium bohemicum S15	ND	Ga0077217_102148	Ga0077217_103872	Ga0077217_120586, 120587	ND
Acidobacterium ailaaui PMMR2	ND	N655DRAFT_2950	N655DRAFT_2813	N655DRAFT_0140, 0141	ND
**Subdivision 3**					
"Candidatus Solibacter usitatus" Ellin6076		Acid_1079, 3468	Acid_2824	Acid_1871, 1872, 6987, 6988, 6989, 6990, 7692, 7693	Acid_6996

Acidobacteria bacterium KBS 96	G004DRAFT_5151 & 5153	ND	G004DRAFT_2333	ND	ND
Bryobacter aggregatus MPL3	ND	M017DRAFT_1799	M017DRAFT_2772	ND	ND
**Subdivision 4**					
Chloracidobacterium thermophilum B	ND	Cabther_A0695	Cabther_A1903	Cabther_B0326, B0327	ND
Pyrinomonas methylaliphatogenes K22	ND	Ga0098325_108216	Ga0098325_108311	ND	ND
**Subdivision 6**					
Luteitalea pratensis DSM100886	Ga0133452_112042	ND	Ga0133452_114156	Ga0133452_114148, 114149	Ga0133452_114104
**Subdivision 8**					
Holophaga foetida TMBS4	ND	ND	HolfoDRAFT_3426	HolfoDRAFT_0146, 0147, 3365	HolfoDRAFT_0066, 0241, 0242, 0243, 0244
Geothrix fermentans DSM14018	ND	ND	G398DRAFT_00282	G398DRAFT_00715, 00716, 02143, 02144, 02484	G398DRAFT_01330
**Subdivision 23**					
Thermoanaerobaculum aquaticum MP-01	ND	EG19_12120	EG19_08945	EG19_09710, 09715, 09720, 09725, 09950	EG19_02555, 02570,04855

"ND" – not detected.

Table S9. Locus tag for the putative genes along with genome ID identified across the acidobacterial genomes associated with the group 1h/5 [NiFe]-hydrogenases. Locus tags for each gene starts with "locus tag start", followed by the number in the respective column.

Gene name	*Acidobacteriaceae bacterium* KBS 83 (G002DRAFT)	*Edaphobacter aggregans* DSM19364 (Q363DRAFT)	*Granulicella mallensis* MP5ACTX8 (AciX8)	*Acidobacteria bacterium* KBS 96 (G004DRAFT)	*"Ca.* Solibacter usitatus" Ellin6076 (Acid)	*Pyrinomonas methylaliphatogenes* K22 (Ga0098325)
Catalytic subunits						
hhyS	0345	01013	4213	3346	6923,6925	108803
hhyL	0346	01012	4214	3345	6926	108804
HP				3344		
Unknown proteins						
FeS protein	03437	01011	4215	3343	6924	108805
HP	03438	01010	4216	3342	ND	108806
HP	03439	01009	4217	3341	ND	108807
HP	03440	01008	4218	3340	ND	108808
HP	ND	ND	4219	ND	ND	108809
Maturation protease						
HupD	03441	01007	4220	3339	6927	108810
hypABCDEF, hydrogenase maturation proteins						
HypA	03448	01000	4222	3331	6923	108813
HypB	03449	00999	4223	3330	6932	108814
HypC	03442	01006	4210	3337	6928	108815
HP	ND	ND	4221	ND	6933	108816
HypD	03443	01005	4211	3336	6929	108817
HypE	03444	01004	4212	3335	6930	108818
GmhA	03445	01003	ND	3334	ND	108819
GrxC	03446	01002	ND	3333	ND	108820
HypF	03447	01001	4209	3332	6931	108821

ND = indicates either the gene was not identified.

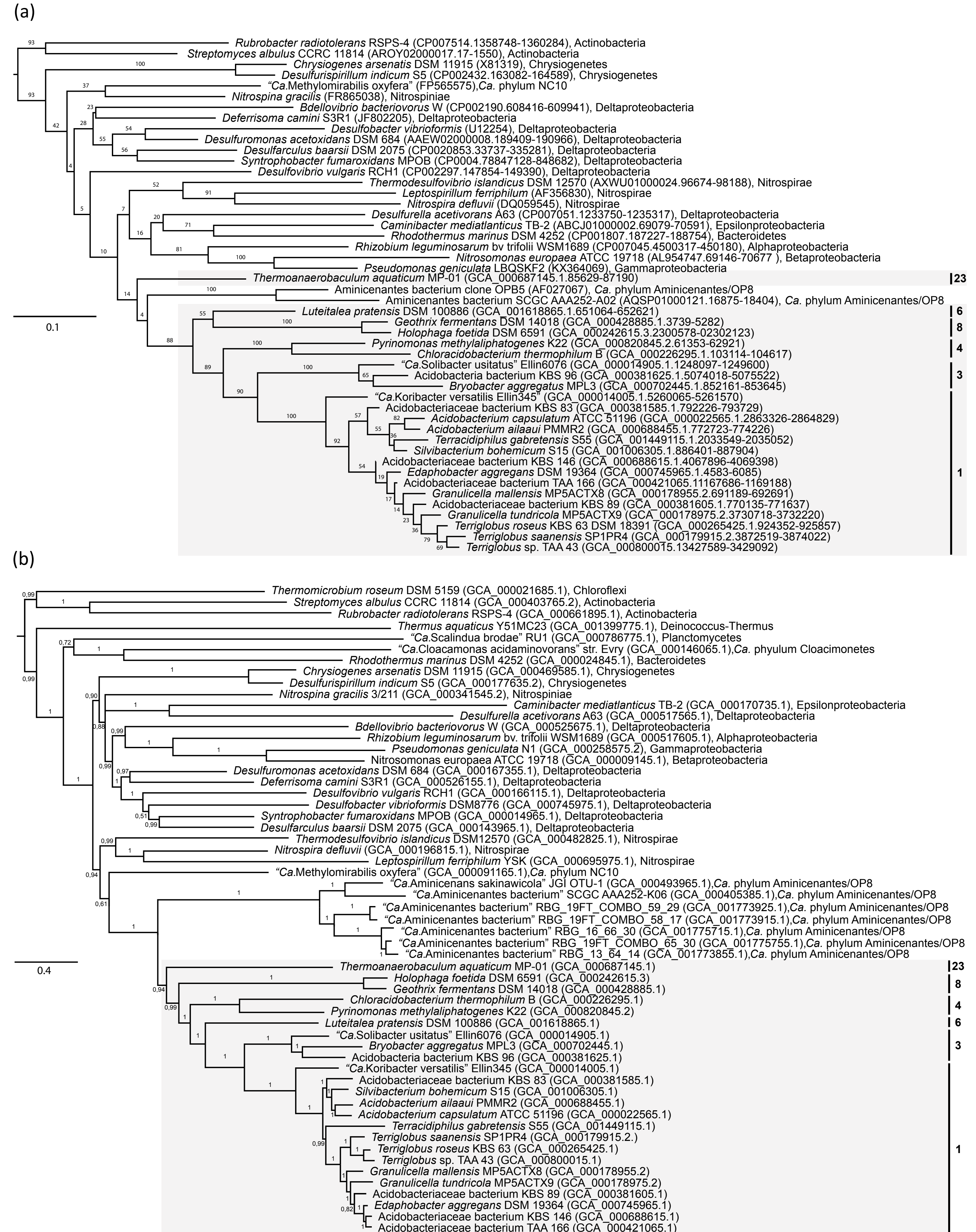

Fig. S1. Panel a depicts an acidobacterial 16S rRNA gene phylogenetic tree (ca. 1,248 nucleotides) of genomes retrieved in this study (8 taxa) and from publicly available genomes of cultivated strains (16 taxa), as inferred by maximum likelihood (RAxML), using the general time-reversible substitution model under the gamma model of rate heterogeneity (GTRGAMMA). Bootstrap support values (1000 iterations) are given on the branches of the tree. Genome assembly- and accession numbers, coordinates and accession numbers of the 16S rRNA genes are given in brackets. Numbers to the right of the tree correspond to the acidobacterial subdivisions. Scale bar indicates estimated nucleotide substitution per site. The root was placed on the branch leading to the *Actinobacteria*, belonging to the *Terrabacteria*. Panel b depicts the comprehensive phylogenomic tree (the part marked in grey is shown in Figure 2) by Bayesian inference based on a concatenated dataset of 43 universally conserved marker genes. The scale bar indicates 0.4 changes per nucelotide. Additional details can be found in Supporting Information.

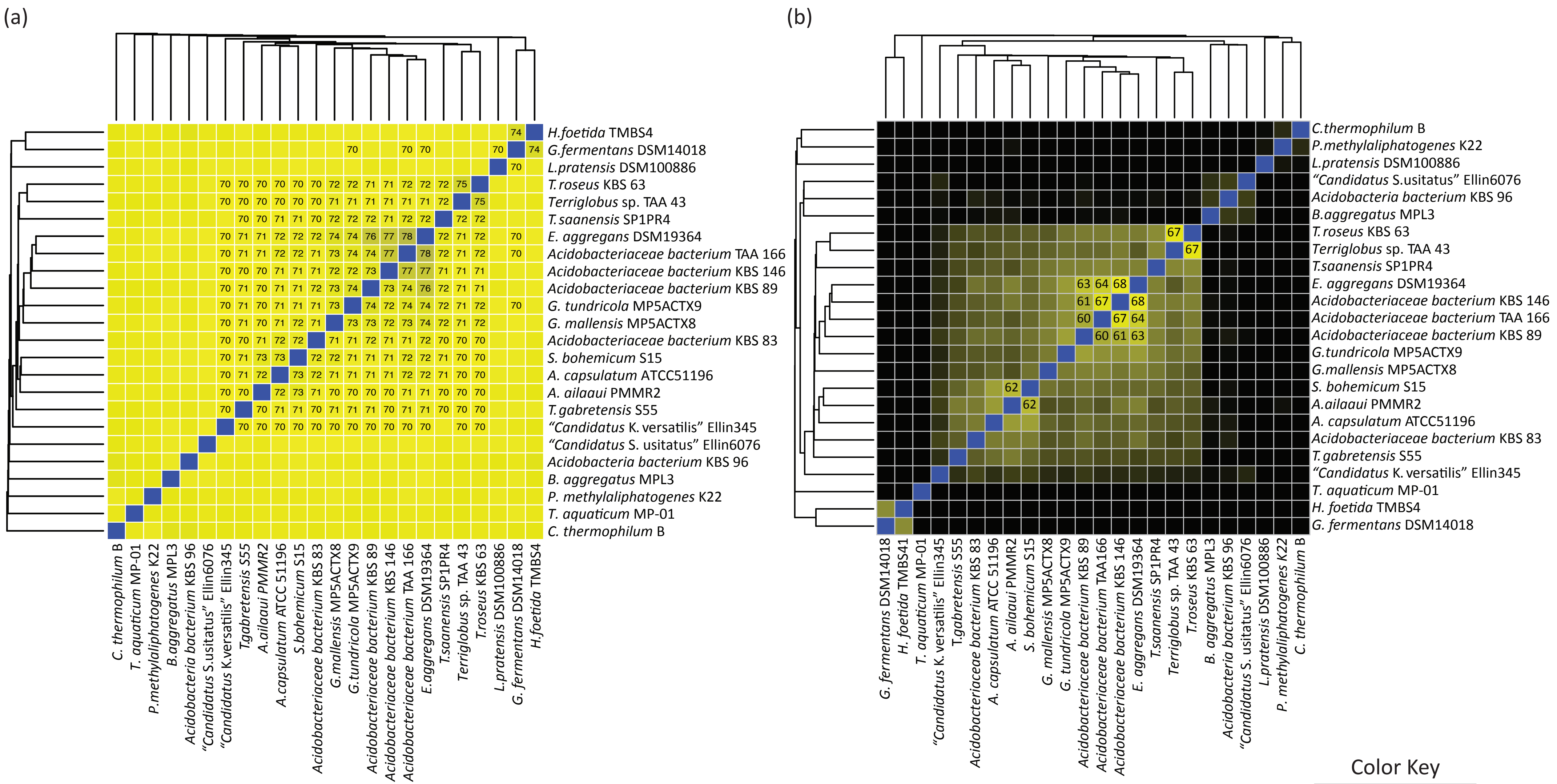

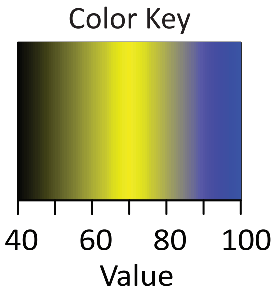

Fig. S2. Average nucleotide identity (panel a) and average amino acid identity (panel b) clustering analysis across the acidobacteria genomes. The heatmap was generated in the R package (ggplot2). Color scale bar represents percent identity.

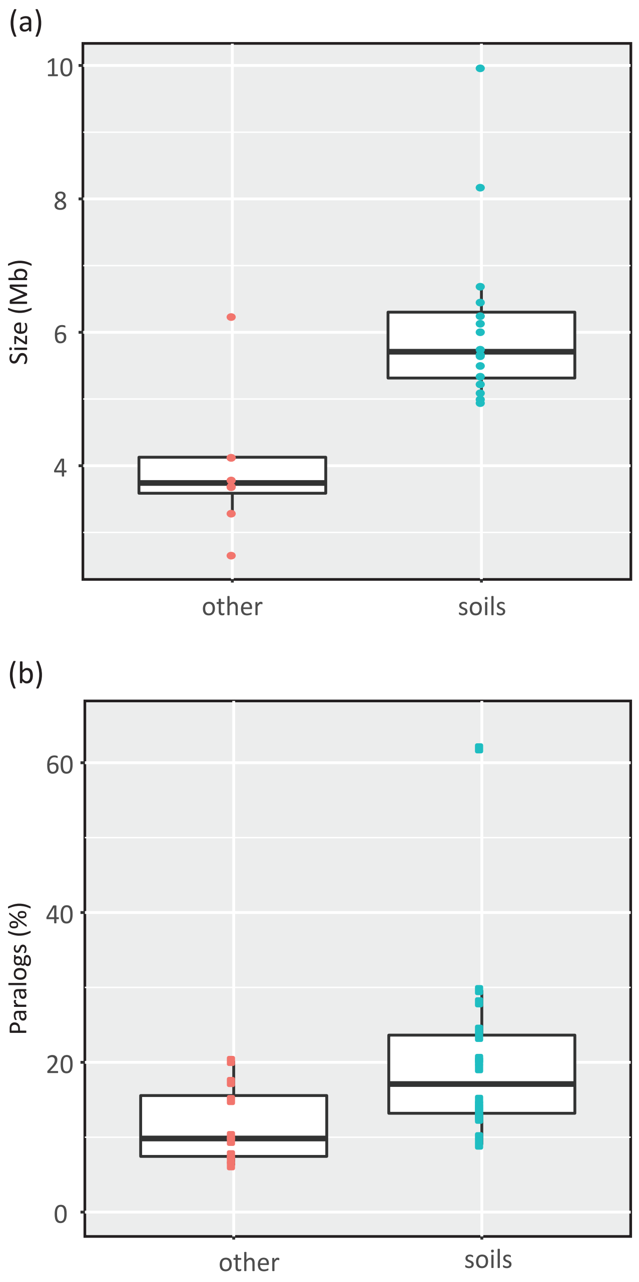

Fig. S3. Distribution of genome size (panel a) and percent of paralogous genes (panel b) across genomes stemming from “soils” and “other” environments. The percent of paralogous genes were normalized to gene count. The definition of “soil” vs “other” genomes can be found in Table S1. Data were obtained from the IMG JGI website.

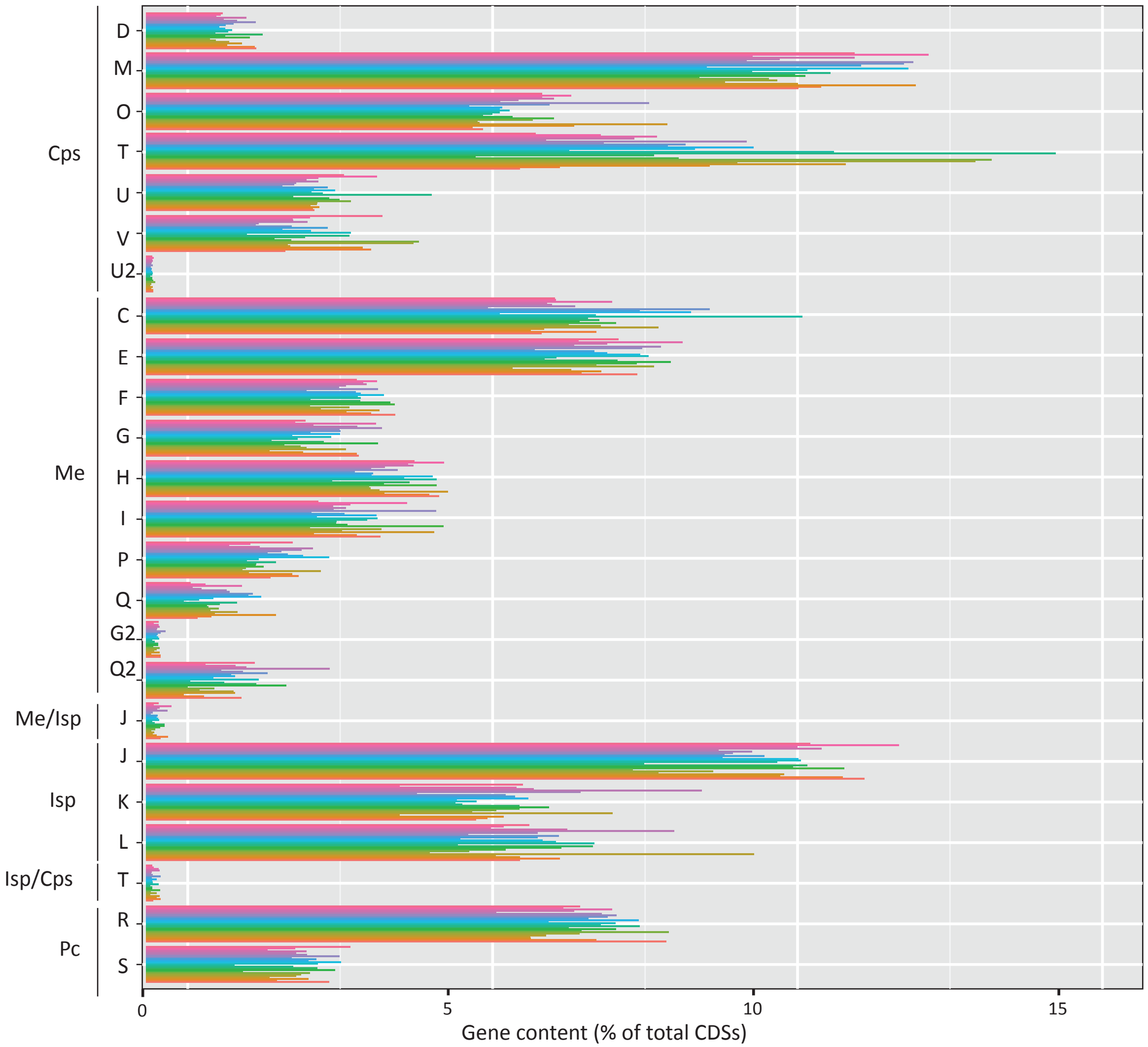

Cellular processes and signaling (Cps)
[D] Cell cycle control, cell division, chromosome partitioning
[M] Cell wall/membrane/envelop biogenesis
[O] Posttranslational modification, protein turnover, chaperones
[T] Signal transduction mechanisms
[U] Intracellular trafficking, secretion, & vesicular transport
[V] Defense mechansims
[U2] Cell motility, posttranslational modification, protein turnover, chaperones/intracellular trafficking, secretion, & vesicular transport

Metabolism (Me)
[C] Energy production and conversion
[E] Amino acid transport and metabolism
[F] Nucleotide transport and metabolism
[G] Carbohydrate transport and metabolism
[H] Coenzyme transport and metabolism
[I] Lipid transport and metabolism
[P] Inorganic ion transport and metabolism
[Q] Secondary metabolities biosynthesis, transport and catabolism
[G2] Nucleotide transport and metabolism, carbohydrate transport and metabolism
[Q2] Lipid transport and metabolism, secondary metabolites biosynthesis, transport and catabolism

Metabolism/Information storage processing (Me/Isp)
[J] Nucleotide transport and metabolism, translation, ribosomal structure and biogenesis

Information Storage and processing (Isp)
[J] Translation, ribosomal structure and biogenesis
[K] Transcription
[L] Replication, recombination and repair

Information Stroage and Processing/Cellular processes and signaling (Isp/Cps)
[T] Transcription, sigmal transduction mechanisms

Poorly characterized (Pc)
[R] General function prediction only
[S] Function unknown

Fig. S4. Gene content by COG functional categories within the acidobacterial core genome. Each color represents a different genome. Functional categories are grouped by metabolism (Me), cellular processes (Cp), information storage and processing (Isp), and poorly characterized (Pc).

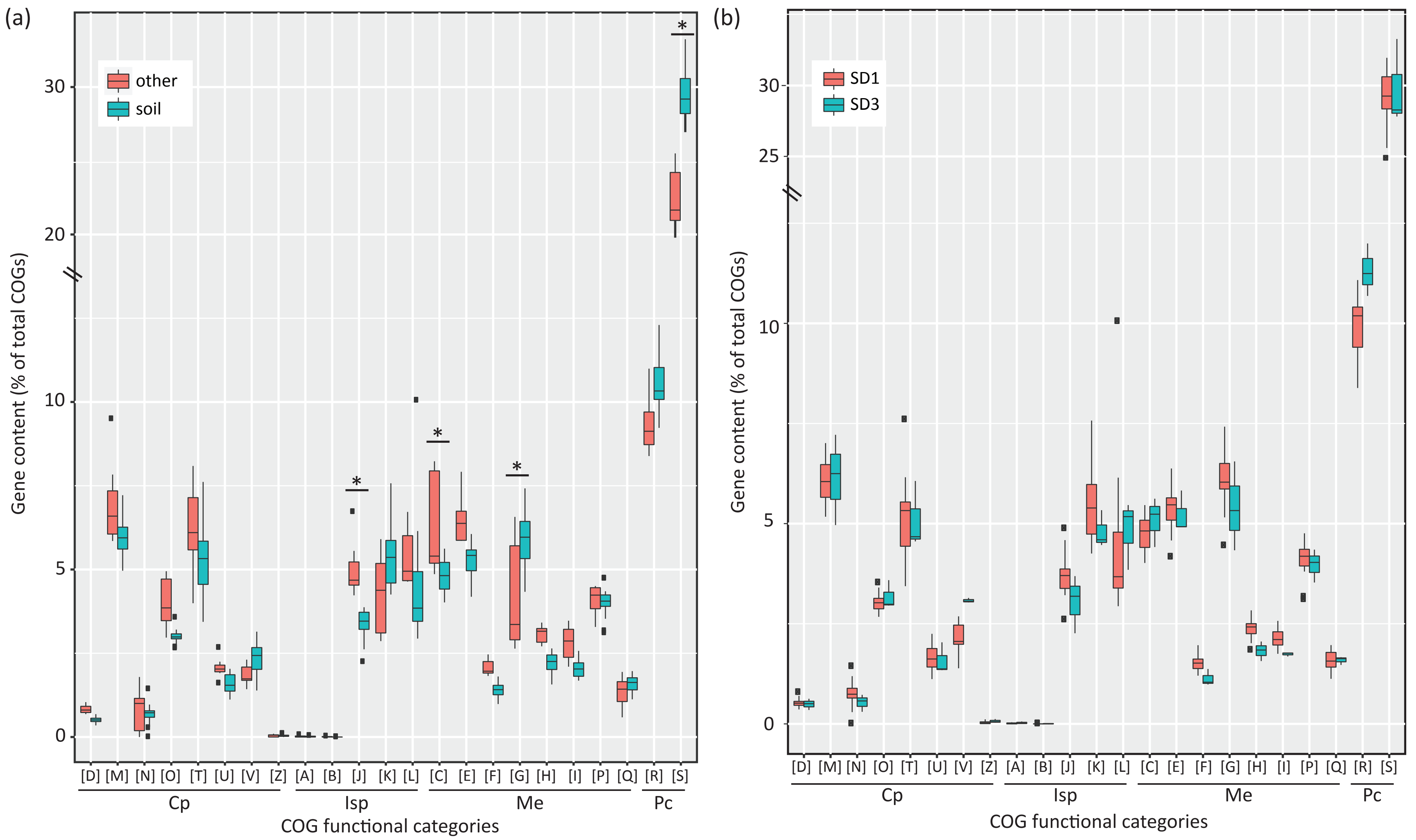

Fig. S5. Boxplot distribution of COG functional categories across environments (panel a) and subdivision 1 and 3 genomes (panel b). COG functional categories are grouped by cellular processes (Cp), information storage and processing (Isp), metabolism (Me) and poor characterized (Pc). The description of the sub-categories can be found in Fig. S3 and definition of “soil” vs “other” genomes can be found in Table S1. Asterisks depict significant differences based on a chi-square goodness of fit.

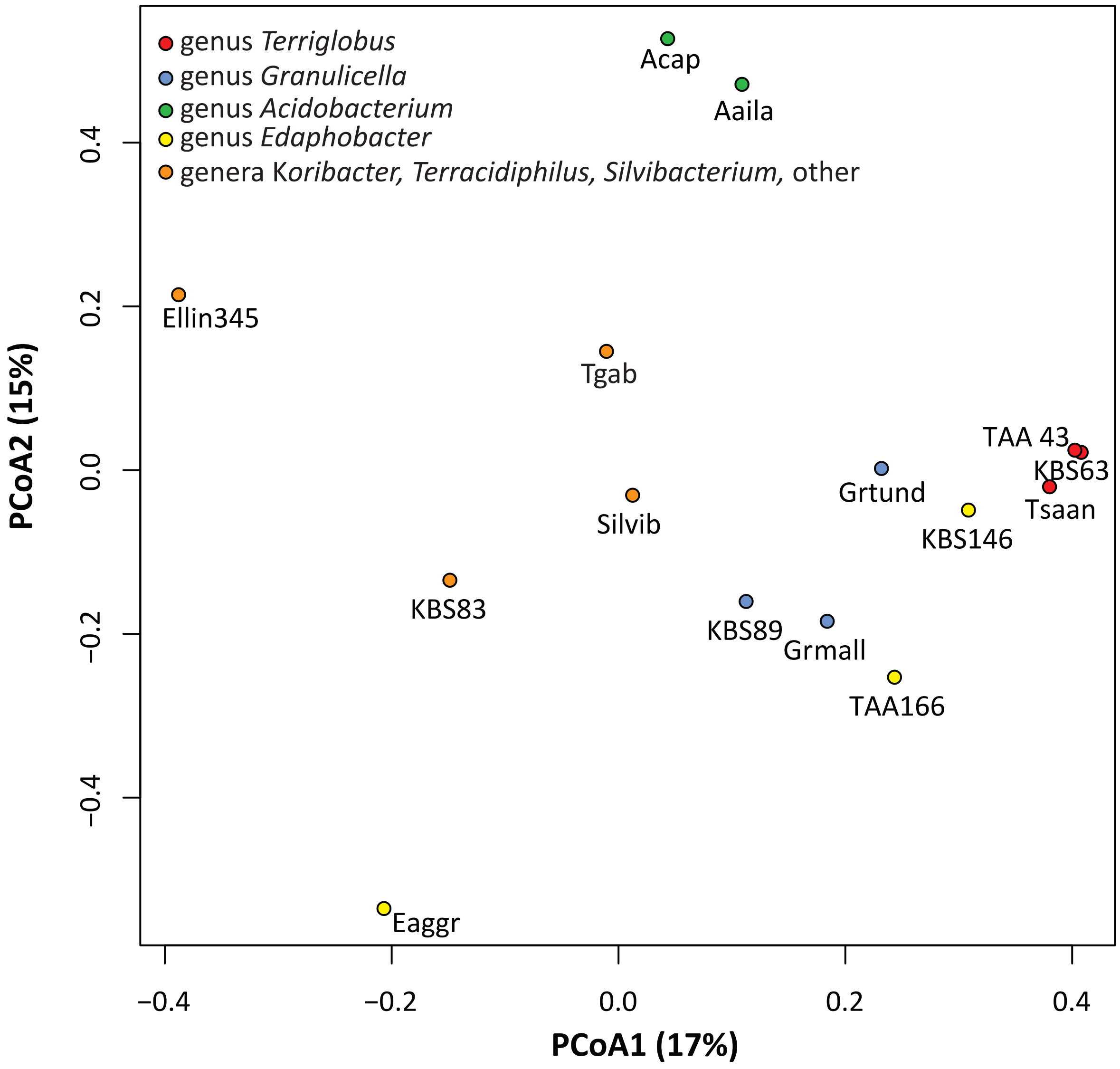

Fig. S6. PCoA plot based on the COG/NOGs of the genomes of genera from subdivision 1 based on the Bray–Curtis distance. The listed abbreviations for the genomes can be found in Table S1. Genera are depicted in different colors.

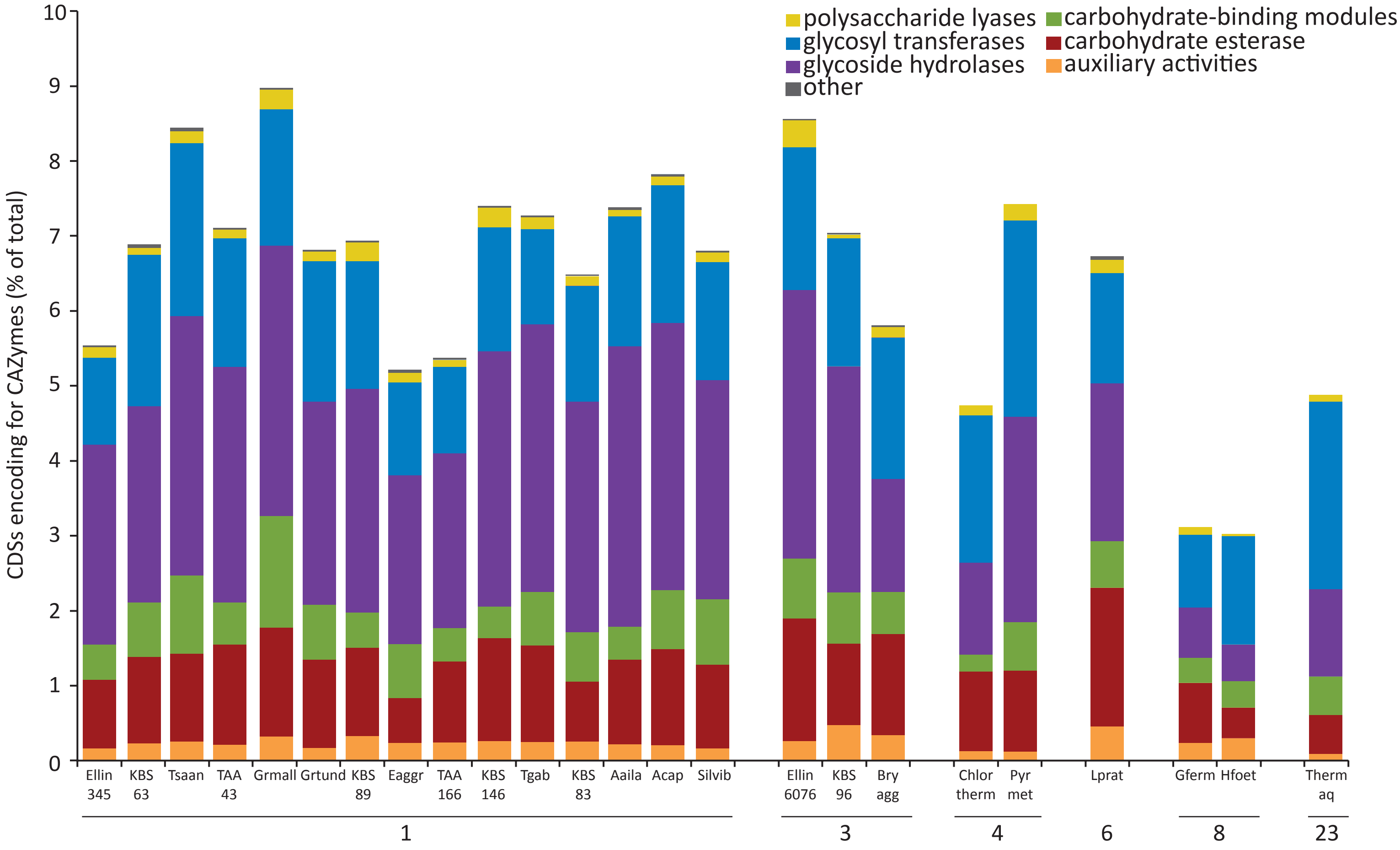

Fig. S7. Distributions of CDSs encoding for carbohydrate-active enzymes based on the database dbCAN (http://csbl.bmb.uga.edu/dbCAN/index.php), specifically depicting polysacharide lyases, glycosyl transferases, glycoside hydrolases, carbohydrate-binding modules, carbohydrate esterase and auxiliary activities. "Other" denotes a sum of CDSs encoding for cohesion, dockerin and S-layer.

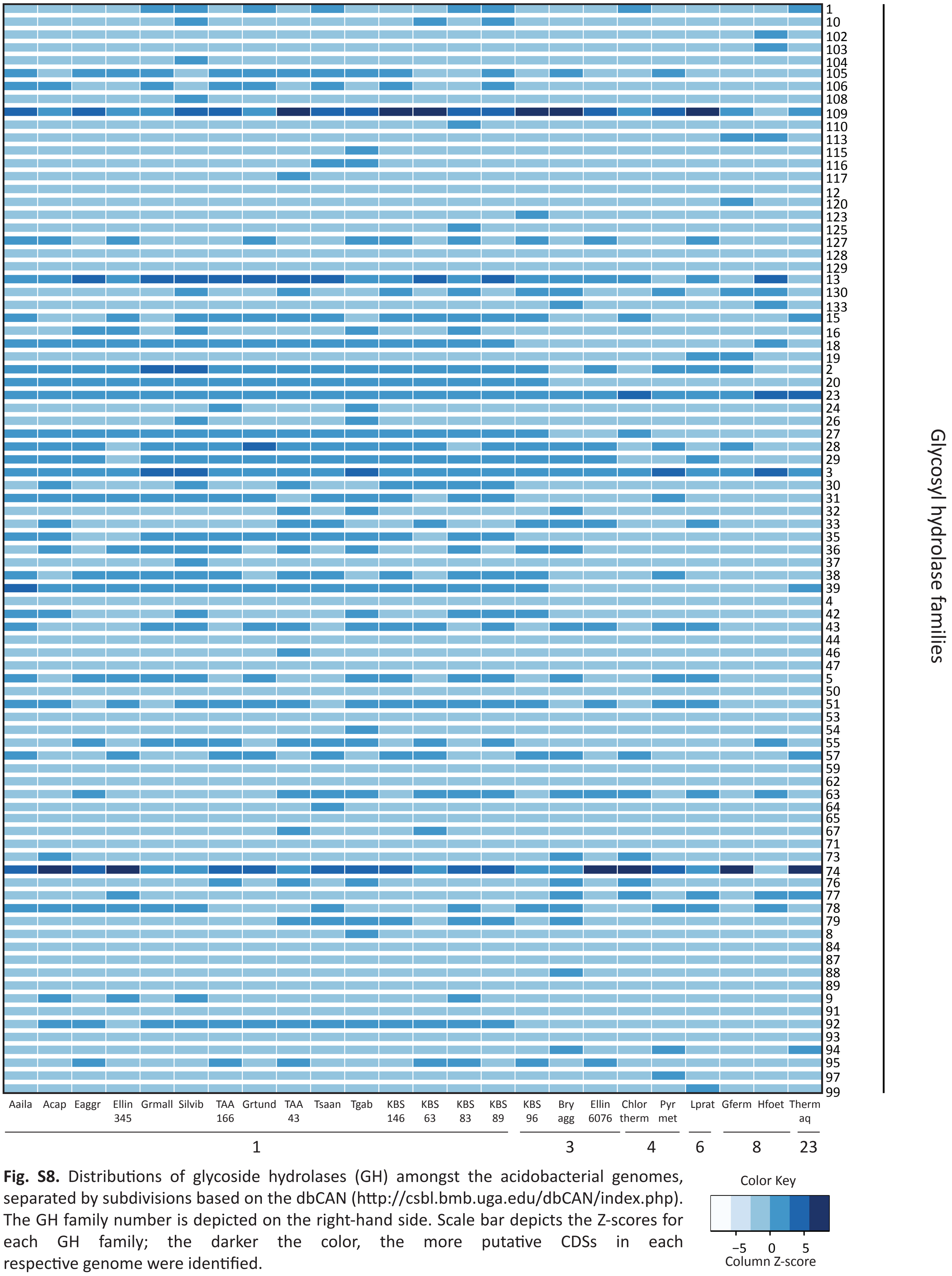

Fig. S8. Distributions of glycoside hydrolases (GH) amongst the acidobacterial genomes, separated by subdivisions based on the dbCAN (http://csbl.bmb.uga.edu/dbCAN/index.php). The GH family number is depicted on the right-hand side. Scale bar depicts the Z-scores for each GH family; the darker the color, the more putative CDSs in each respective genome were identified.

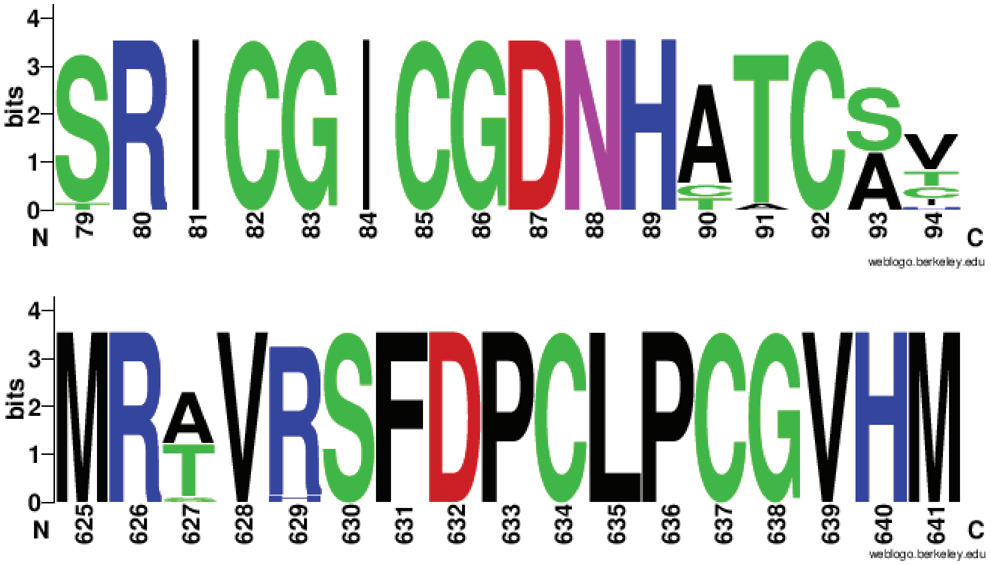

Fig. S9. Consensus L1 and L2 signature in the large subunit of the group 1h/5 [NiFe]-hydrogenase across the 6 acidobacterial strains. Images were generated using WebLogo (http://weblogo.berkeley.edu). Reference: Crooks, G. E., G. Hon, J.-M. Chandonia, and S. E. Brenner. 2004. WebLogo: a sequence logo generator. Genome Res. 14:1188–1190.
